# Effect of eicosapentaenoic acid on innate immune responses in Atlantic salmon cells infected with infectious salmon anemia virus

**DOI:** 10.1186/s12985-024-02619-0

**Published:** 2025-01-09

**Authors:** Ingrid Holmlund, Samira Ahmadi, Bente Ruyter, Tone-Kari Østbye, Marta Bou, Tor Gjøen

**Affiliations:** 1 University of Oslo, Oslo, Norway; 2 Nofima, Ås, Norway

**Keywords:** Atlantic salmon, Polyunsaturated fatty acid, Eicosapentaenoic acid, Virus, Infectious salmon anemia virus, Transcriptomics, Viral disease, Ferroptosis

## Abstract

Aquaculture is one of the world's fastest-growing sectors in food production but with multiple challenges related to animal handling and infections. The disease caused by infectious salmon anemia virus (ISAV) leads to outbreaks of local epidemics, reducing animal welfare, and causing significant economic losses. The composition of feed has shifted from marine ingredients such as fish oil and fish meal towards a more plant-based diet causing reduced levels of eicosapentaenoic acid (EPA). The aim of this study was to investigate whether low or high levels of EPA affect the expression of genes related to the innate immune response 48 h after infection with ISAV. The study includes seven experimental groups: ± ISAV and various levels of EPA up to 200 µM. Analysis of RNA sequencing data showed that more than 3000 genes were affected by ISAV alone (without additional EPA). In cells with increasing levels of EPA, more than 2500 additional genes were differentially expressed. This indicates that high levels of EPA concentration have an independent effect on gene expression in virus-infected cells, not observed at lower levels of EPA. Analyses of enriched biological processes and molecular functions (GO and KEGG analysis) revealed that EPA had a limited impact on the innate immune system alone, but that many processes were affected by EPA when cells were virus infected. Several biological pathways were affected, including protein synthesis (ribosomal transcripts), peroxisome proliferator activated receptor (PPAR) signaling, and ferroptosis. Cells exposed to both increasing concentrations of EPA and virus displayed gene expression patterns indicating increased formation of oxygen radicals and that cell death via ferroptosis was activated. This gene expression pattern was not observed during infection at low EPA levels or when Atlantic salmon kidney (ASK) cells were exposed to the highest EPA level (200 μM) without virus infection. Cell death via ferroptosis may therefore be a mechanism for controlled cell death and thus reduction of virus replication when there are enough polyunsaturated fatty acids (PUFAs) in the membrane.

**Supplementary Information:**

The online version contains supplementary material available at 10.1186/s12985-024-02619-0.

## Introduction

Since the first reports of an Atlantic salmon anemia disease and the identifications of the causative agent, infectious salmon anemia virus (ISAV) [1], this virus has created serious health and fish welfare problems on both sides of the Atlantic [2, 3]. Using strict management procedures, the initial wave of outbreaks was reduced [4] and vaccines have been developed [5], but this disease is still a serious problem for the salmon aquaculture industry. ISAV belongs to the Orthomyxoviridae negative sense segmented RNA viruses which also includes the influenza genera [6]. Detailed studies of host and tissue tropism [2, 7, 8], uptake [9, 10], replication [11, 12] as well as innate [13–15] and adaptive immune responses during ISAV infections [16–19] have been reported but many questions regarding virus-host interactions remains to be investigated. The recent publication of the first reverse genetics system for ISAV will certainly open new avenues for deeper molecular characterization and vaccine development [20]. Another important area of research into virus-host interactions is the role of dietary and cellular fatty acids on innate and adaptive immune responses during infection. Results from both experimental [21–25] and clinical studies [26–29] suggest that polyunsaturated fatty acids (PUFA) like eicosapentaenoic (EPA) and docosahexaenoic acid (DHA) and their metabolites play important roles in host responses against a range of infections [30]. This focus on the interplay between metabolism and immunity have led to development of a branch of immunology called immunometabolism [31] and many of these insights are relevant for aquaculture as much as the feed is the one of the main elements in the production chain of fish [32]. As in mammals, multiple studies suggest a role for PUFAs in the maintenance of growth and health in Atlantic salmon [33–35] as well as immunity [36–38]. To protect limited marine raw materials (like herring and capelin) for salmon feed production, plant and algae based raw materials with lower PUFA levels are taking over [32]. Although long chain PUFAs like EPA and DHA have been regarded as essential for optimal growth and development in vertebrates [33], the dietary demand for EPA in Atlantic salmon has recently been questioned [39]. Recent findings concerning the intersection of energy metabolism with innate immunity to viral infections like the interaction of *STING* (stimulator of interferon genes) with *FADS2* (fatty acid synthase 2) [40] and the role of lactate in regulation of MAVS (mitochondrial antiviral signaling protein) [41] suggest that dietary lipids play a role in innate immunity. Likewise, expression levels of an enzyme involved in production of endogenous fatty acids (oleoyl-acyl-carrier-protein (ACP) hydrolase (OLAH)) was associated with severity of multiple viral respiratory functions via effects on macrophage lipid droplet dynamics [42]. The regulated cell death pathway named ferroptosis [43] occurring during various forms of viral infections [44] have also been linked to the level of cellular PUFAs. These recent developments incited us to investigate the role of EPA in antiviral immunity in Atlantic salmon kidney (ASK) cells. The cellular levels of EPA may affect antiviral signaling responses in at least three separate ways. Firstly, as ligands for peroxisome proliferator-activated receptors (PPARs) or G-protein coupled receptor 120 (GPR120) [45]. Secondly, as metabolic precursors of immune modulators like resolvins and eicosanoids [46] and lastly, by altering the composition of membrane microdomains called “rafts” where membrane bound signaling proteins like toll-like receptors (TLRs) and MAVS anchor and signal from [47, 48]. Previous studies of PUFA effects on innate immunity in tissues or cells from Atlantic salmon are not conclusive as EPA may confer detrimental [33, 49], neutral, [50, 51] or supportive [52] effects, depending on developmental stage and type of stressor. To gain a more mechanistic view of the interplay between EPA and innate immunity to viral infection in Atlantic salmon, we measured transcriptional responses to ISAV infection at five different cellular EPA levels. One of the main findings not observed with virus or high EPA alone (only in combination) were the enrichment of transcripts related to the ferroptosis and PPAR pathways. This may suggest that the combined stress of high PUFA and viral infection initiates iron dependent lipid peroxide formation and cell death as a host defense mechanism to control viral replication.

## Materials and methods

### Cell culture

Knut Falk (Norwegian Veterinary Institute) kindly provided the Atlantic salmon kidney (ASK) cell line used in this project. The cells were cultivated at 20 °C and split (1:2) once a week. The cell media consisted of Leibovitz L-15 medium (Lonza BioWhittaker, Verviers, Belgium) supplemented with L-glutamine (4 mM—Lonza BioWhittaker, Verviers, Belgium), fetal bovine serum (10%—Gibco, Life Technologies, Bleiswijk, The Netherlands), 2-mercaptoethanol (40 µM—Gibco, Life Technologies, Bleiswijk, The Netherlands) and gentamicin (50 mg/mL—Lonza BioWhittaker, Walkersville, USA).

The cells were acclimatized one week before the experiment started; the cultivation temperature was reduced to 15 °C, and the content of fetal bovine serum in the media was reduced to 2%. These conditions were also used during the experimental period.

### Virus propagation

The ISAV strain used in this experiment was Glesvær 2/90, which has been shown to result in high mortality in Atlantic salmon [53]. The virus was produced and isolated as described by Andresen et al. [54].

### Experimental design

ASK cells (passages 40–50) were seeded in 14 wells (35 mm, 6-well plates), with a density of 1.5 × 10^5^ cells per well. The cells were cultivated overnight (15 °C) for adhesion. Thereafter, EPA (Sigma-Aldrich, St. Louis, MO, USA) (bound to BSA) of different concentrations (0, 25, 50, 100, 200 µM) was added to the wells (*n* = 2 for 25, 50 and 100 µM, *n* 4 for 0 and 200 µM), and the cells were incubated for 7 days. The wells were washed three times with sterile PBS (QIAGEN, Hilden, Germany), before performing the in vitro infection.

A virus suspension with a multiplicity of infection (MOI) of 1 in serum-free L-15 medium was added to 10 of the wells (0, 25, 50, 100, 200 µM). The extra wells without EPA (*n* = 2) and with 200 µM EPA (*n* = 2) were used as uninfected controls. The infected wells were incubated for 4 h to allow for virus adsorption, followed by addition of previous culture medium (L-15 supplemented ± EPA).

The cells were incubated for 48 h post-infection (9 days of cultivation in total), then washed three times with PBS, lysed using buffer RLT (QIAGEN, Hilden, Germany) and stored at −20 °C until RNA isolation. This experiment was repeated three times, which resulted in six technical replicates per sample (*n* = 6), and 42 samples in total.

### Fatty acid analysis

ASK cells were grown in flasks (75 cm^2^) with L-15 supplemented medium (2% FBS) and EPA (0, 25, 50, 100, 200 µM) for one week. The total lipids of the cells and cell culture media were extracted as described by Folch et al. [55], by homogenizing the tissue with 2:1 chloroform–methanol (v/v). The chloroform phase was isolated, and nitrogen was used to evaporate the solvent, resulting in the residual lipid extract. Benzene was used to re-dissolve the lipids, and 2,2-dimethoxypropane and methanolic HCl were added for transesterification overnight at room temperature, as described by Mason and Waller and by Hoshi et al. [56, 57]. A gas chromatograph (Hewlett Packard 6890) with helium as a carrier gas, a split injector, a SGE BPX70 capillary column (length: 60 m, internal diameter: 0.25 mm, thickness of film: 0.25 µM), a flame ionization detector (FID) and the HP Chem Station software was used to separate the fatty acid methyl esters and monitor the process. The detector and injector of the chromatograph had a temperature of 300 °C, while the oven temperature was raised from 50 to 170 °C (4 °C /min) and then further raised to 200 °C (0.5 °C /min).

Peaks appeared in the chromatogram as the different compounds eluted from the column and passed through the detector. Individual fatty acid methyl esters were identified by reference to well-characterized standards. The relative amount of each fatty acid was expressed as a percentage of the total amount of fatty acid in the analyzed sample, and the absolute amount of fatty acid per gram of tissue was calculated using C23:0 methyl ester as the internal standard.

### Total RNA isolation

Total RNA was extracted for sequencing and qPCR using RNeasy Mini Kit (QIAGEN, Hilden, Germany) following the manufacturer’s tissue protocol, with an optional on-column DNase I digestion to remove gDNA (RNase-Free DNase Set, QIAGEN, Hilden, Germany). The RNA samples were eluted in 50 µL RNase free distilled water, and the RNA concentrations were measured using a PicoDrop Pico100 (PicoDrop Technologies, Cambridge, UK).

### RNA sequencing

The RNA samples (*n* = 42) were sent to the Norwegian Sequencing Centre (NSC). The RNA qualities were checked using the Agilent 2100 Bioanalyzer (Agilent, Santa Clara, USA), confirming sample quality (RIN > 8) and purity (no additional peaks). The cDNA libraries were prepared using the TruSeq Stranded mRNA Library Prep Kit (Illumina Inc., San Diego, USA) and sequenced to 150 bp paired end reads with the Illumina HiSeq 4000 sequencer.

### Qualitative PCR (qPCR)

The High-Capacity cDNA Reverse Transcription Kit (Applied Biosystems, United States) was used to make cDNA, using the manufacturer’s protocol. The LightCycler 480 and SYBR Green Master Mix (both from Roche Diagnostics, Basel, Switzerland) was used to perform qPCR in 96-well plates. The initial heating lasted for 5 min (95 °C), and the cycling conditions (40 cycles) were 95 °C (10 s), 60 °C (10 s), and 72 °C (10 s), and the melting curves were measured at 95 °C (5 s) and 65 °C (1 min). The qPCR experiment was repeated three times with two technical replicates per sample. The cycle threshold (Ct) values of the samples were obtained to calculate the relative expression levels of the genes (delta-delta Ct method) [58], with *18S* and *ef1a* as reference genes [59]. The primers used are listed in Table 1.

**Table 1:** List of primers used in qPCR analysis

Genes	Direction	Sequence 5′ → 3′	Accession number	Amplicon	Melting temp [°C]	References
*ef1a*	F	CACCACCGGCCATCTGATCTACAA	AF321836	77	58.8	[60]
	R	TCAGCAGCCTCCTTCTCGAACTTC			60.6	
*18S*	F	TGTGCCGCTAGAGGTGAAATT	AJ427629.1	61	54.8	[61]
	R	GCAAATGCTTTCGCTTTCG			52.9	
*ifna1*	F	CCTGCCATGAAACCTGAGAAGA	AY216594	107	55.4	[59]
	R	TTTCCTGATGAGCTCCCATGC			55.1	
*isg15*	F	ATGGTGCTGATTACGGAGCC	AY926456	151	54.2	[62]
	R	TCTGTTGGTTGGCAGGGACT			53.9	
*mx1/2*	F	TGATCGATAAAGTGACTGCATTCA	NM_001123690.1/ NM_001123693.1	80	54.8	[63]
	R	TGAGACGAACTCCGCTTTTTCA			55.9	
*ifih1*	F	GAGAGCCCGTCCAAAGTGAA	XM_014164134	389	53.9	[54]
	R	TCCTCTGAACTTTCGGCCAC			53.9	
*ISAVseg5*	F	GAAAGCCCTGCTCTGGC	HQ259675.1	50	51.8	[64]
	R	TCCTCAAGTCTGCTTCGGGA			55.2	
*ISAVseg6*	F	AGGCCAAAAACGGAAATGGA	HQ259676.1	118	51.6	[64]
	R	CCGTCAGTGCAGTCATTGGTT			54.9	
*ISAVseg7*	F	GAAATGGACAGAGACGGCGTATCA	HQ259677.1	124	57.9	[64]
	R	GCTCAACTCCAGCTCTCTCATTGT			59.0	

*ef1a*—elongation factor 1 alpha, *18S*—18S ribosomal RNA, *ifna1* – interferon alpha-1, *isg15*—interferon-stimulated gene 15, *mx1/2*—interferon-induced GTP-binding protein Mx1/2, *ifih1*—interferon induced with helicase C domain 1, *ISAVseg5* – infectious salmon anemia virus gene segment 5, *ISAVseg6* – infectious salmon anemia virus gene segment 6, *ISAVseg7* – infectious salmon anemia virus gene segment 7

### Bioinformatics and statistics

HISAT2 [65] was used to map the FASTQ sequence read files from the RNA sequencing to the Atlantic salmon genome (GCF_000233375.1_ICSASG_v2_genomic.fna). The existing Atlantic salmon annotation file (GCF_000233375.1_ICSASG_v2_genomic.gff) was used to assemble the transcripts with StringTie [66]. Both the genome and the annotation file were downloaded from NCBI (annotation release 100). Following the alignment and assembly of transcripts, the R software package DESeq2 (version 1.40.2) was utilized to quantify the differential expression of genes between the samples and against the controls [67, 68]. The resulting gene expression tables were filtered using a threshold of median > 10, to remove genes that had zero or low counts. The adjusted p-value (padj) was calculated using the Benjamini-Hochberg (BH) procedure [69], and the genes classified as differentially expressed genes (DEGs) had a p-value (padj) below 0.01. A gene was considered upregulated if the log2 fold change (Log2FC) > 1, and downregulated if Log2FC < −1. The sample analysis and the exploratory plots are shown in Supplementary file 1. The gene ontology and KEGG pathway analysis was performed using the R package clusterProfiler [70], with 0.01 as a cutoff for the p-values and q-values (BH adjusted). The KEGG pathway maps were visualized using the R package Pathview [71].

## Results

### Verification of infection

**Fig. 1:**
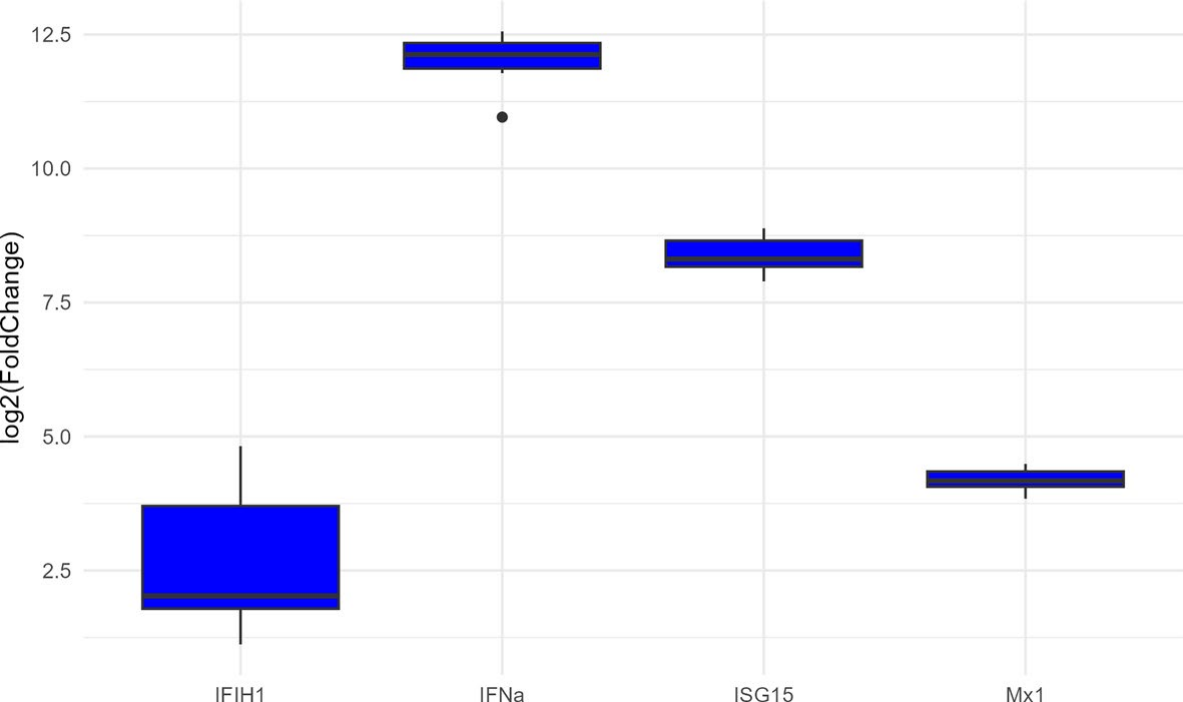
Relative expression of interferon induced with helicase C domain 1 (IFIH1), interferon alpha-1 (IFNa), interferon-stimulated gene 15 (ISG15) and interferon-induced GTP-binding protein Mx1 (Mx1) in infected control vs. non-infected control (0 µM EPA). Expression level is calculated as relative expression to two housekeeping genes (ef1a—elongation factor 1 alpha and 18S—18S ribosomal RNA) using the delta-delta Ct method. Data are displayed as median (horizontal line), 25 and 75% percentiles (box) and 5 and 95% percentiles (whiskers) log2 fold change. All four transcripts were significantly different from control (Wilcoxon rank sum test, *p* < 0.01, *n* = 6)

To verify that cells were infected and responsive before submitting the samples for sequencing, we analyzed a few well known viral and interferon induced salmon transcripts by qPCR [54]. Figure 1 shows that all four transcripts were robustly upregulated by ISAV at 48 h p.i. suggesting that the cells were indeed infected. We also confirmed infection by qPCR of three ISAV genomic RNA segments, and the cells were positive for all (not shown). Figure S8 shows cellular morphology at various time points after infection with ISAV. The cytopathic effect begins to appear at day 3, so RNA samples were isolated at day 2 to avoid the presence of too many dead cells in the samples.

### Fatty acid analysis

The EPA content (%) of cells and cell culture media is presented in Supplementary file 1 (Fig. S1). The cellular content of EPA increased from 2% when no EPA was added, to 21% of total fatty acids when EPA was supplemented at 200 µM.

### RNA sequencing

An exploratory analysis of the RNA-seq raw data is presented in Supplementary file 1 (Fig. S2-S7). Briefly, the samples were sequenced to a depth of about 15–20 million reads (150 nt, double reads) with a mapping frequency to the Atlantic salmon genome of about 80%. Counts were log normally distributed and tests of replicate correlation showed good agreement between technical replicates. PCA and clustering analysis suggested that ISAV infection was the main driver of variability, but EPA also displayed an effect. One of the samples had corrupted sequencing files and were removed from further analyses (EPA, 50 µM, replicate 6).

### Transcriptome effects of ISAV infection and EPA alone

**Fig. 2:**
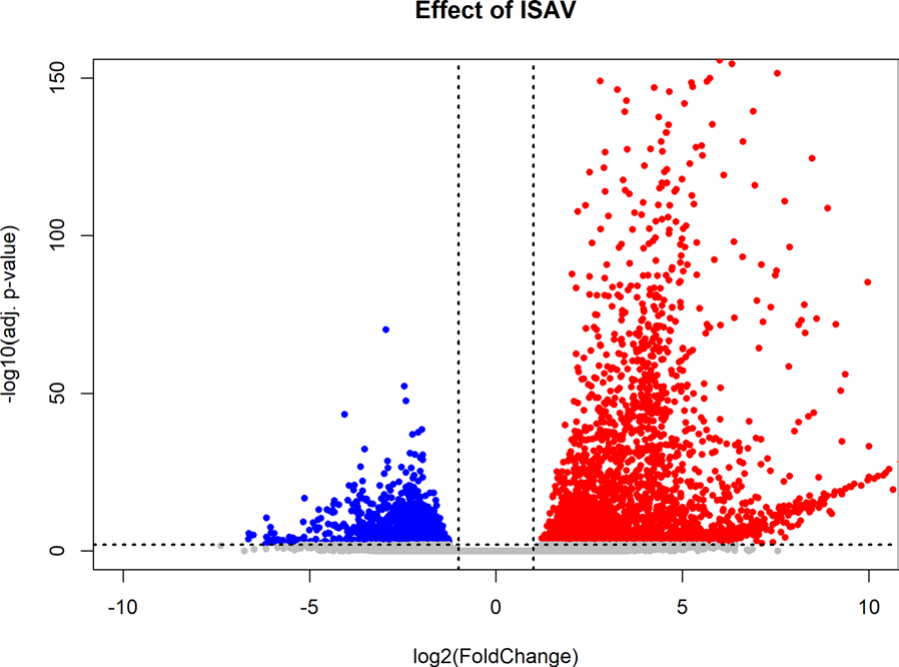
Volcano plot of DEGs in ASK cells 48 h after infection with ISAV. Red dots are upregulated (2090 transcripts), blue dots are downregulated genes (1331 transcripts). Grey dots are not significantly changed (adjusted *p*-value > 0.01)

As previously shown by Andresen [54], infection of ISAV in these cells had strong transcriptional effects with more than 3000 genes dysregulated at 48 h p.i. (Fig. 2).

**Fig. 3:**
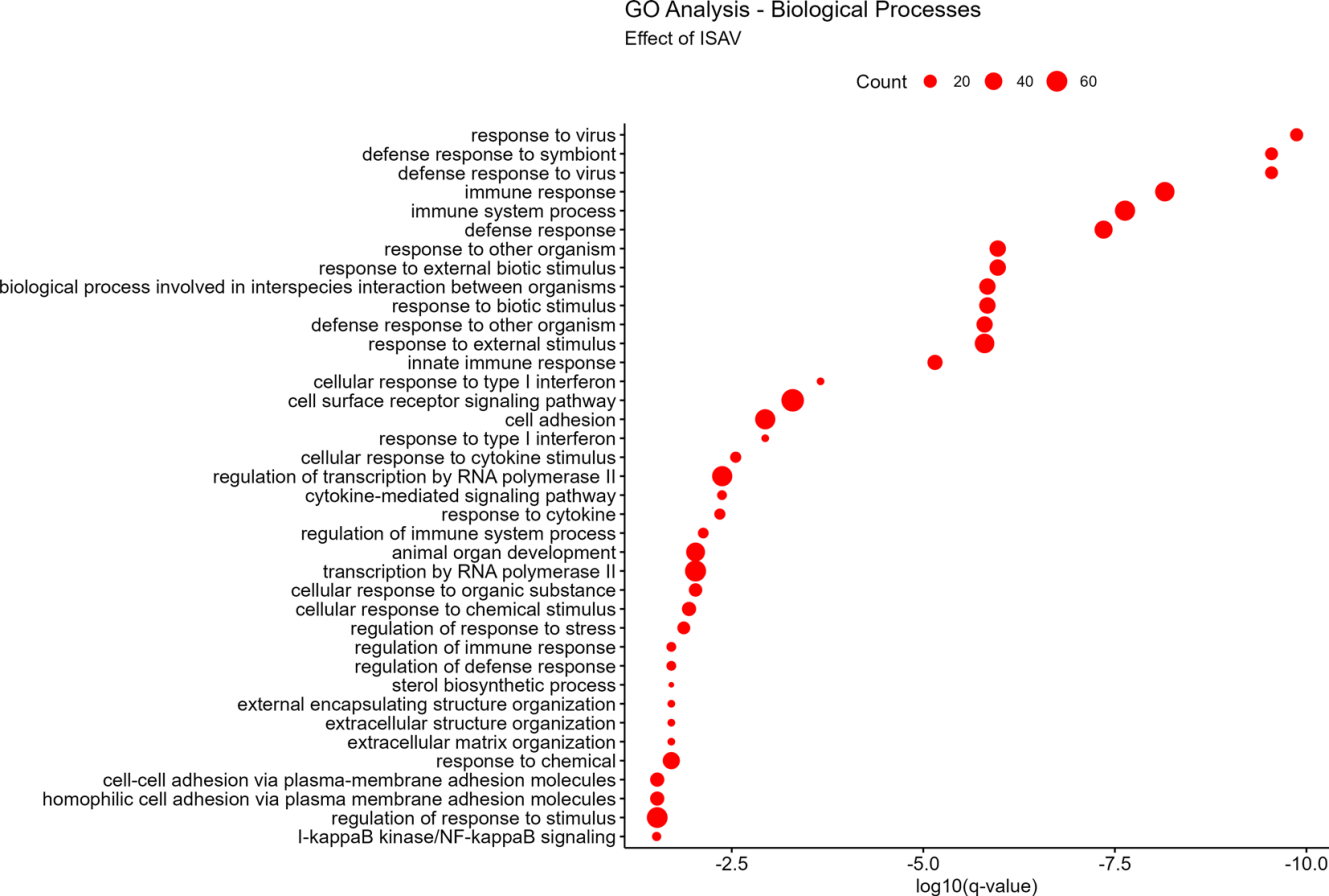
GO enrichment analysis of DEGs in ISAV infected ASK cells

This virus elicits expression of transcripts that were enriched in antiviral and immune system responses related to biological processes and pathways (Fig. 3). All significantly affected genes are listed in Supplementary 2.

As a control, the effects of only 200 µM EPA for 9 days without virus was also analyzed. Only 268 transcripts were moderately differentially expressed in this group, suggesting that EPA treatment alone did not stress the cells (not shown).

### Transcriptome effects of increasing levels of EPA during ISAV infection

**Fig. 4:**
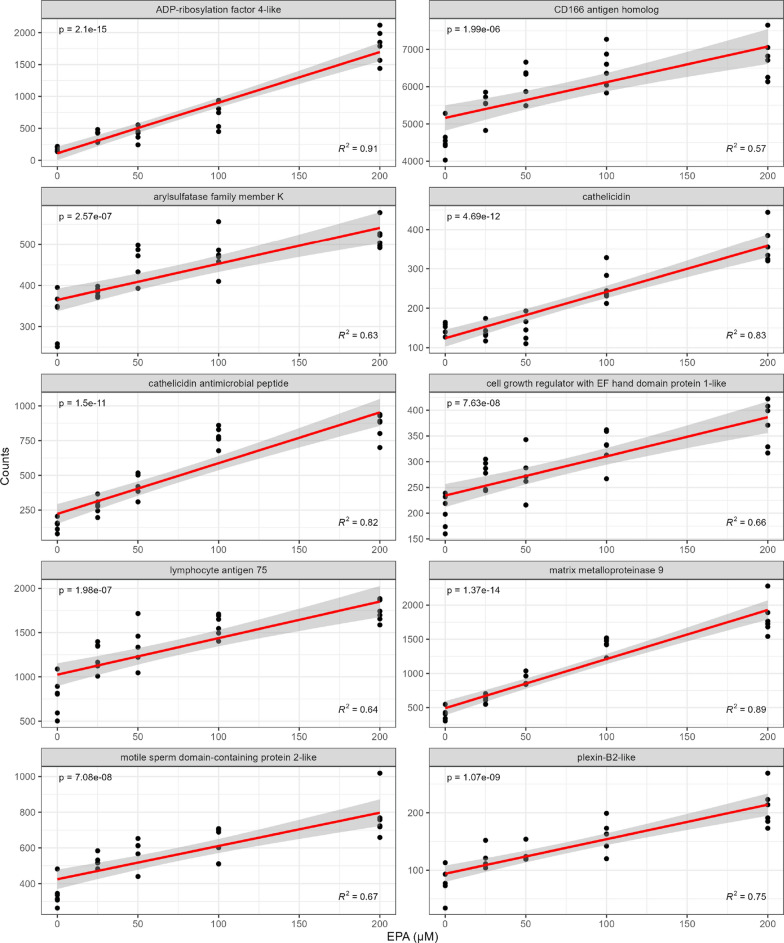
Plot of raw counts and best fit line for the transcripts most positively correlated to EPA concentration in ISAV infected ASK cells

**Fig. 5:**
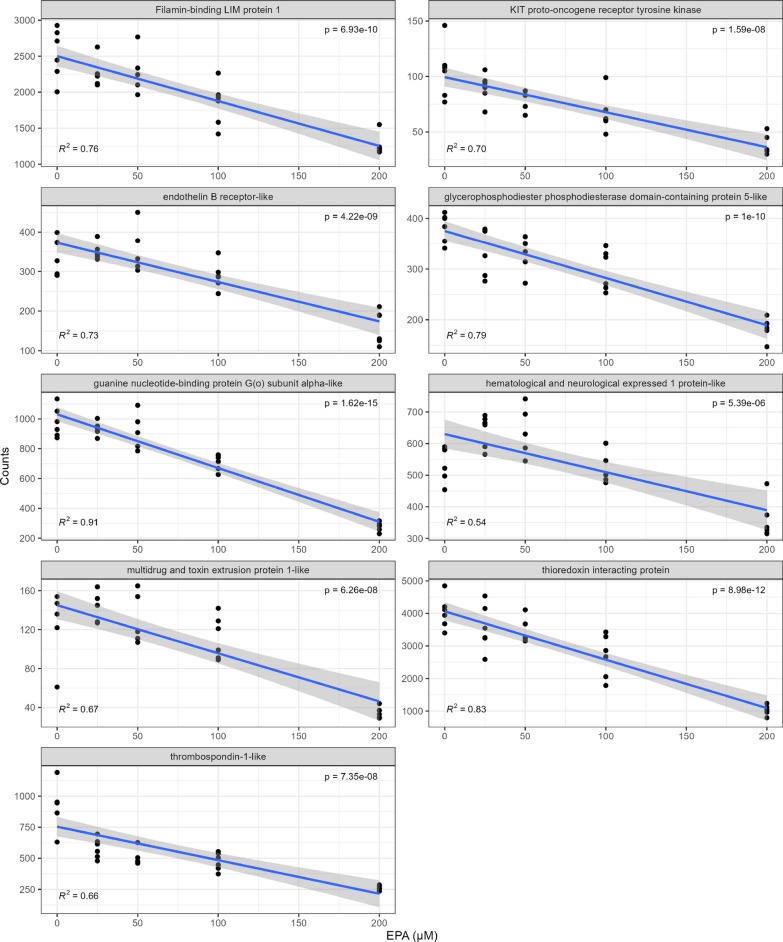
Plot of raw counts and best fit line for the transcripts most negatively correlated to EPA concentration in ISAV infected ASK cells

When cells exposed to increasing levels of EPA (0–200 µM) for 7 days were infected with ISAV, a total of 2921 transcripts were affected by the levels of this fatty acid (adjusted p-value < 0.01) (Supplementary 2). Compared to the effects of virus alone, the changes in expression levels were modest but many transcripts displayed a clear dose response to EPA levels (Figs. 4, 5). Transcripts that showed a positive correlation to EPA levels included antimicrobial peptides (cathelicidin), *MMP9*, and *ARF4* among others. Transcripts with a negative correlation to cellular EPA levels included GTP binding signaling proteins, thrombospondin, and thioredoxin interacting protein.

**Fig. 6:**
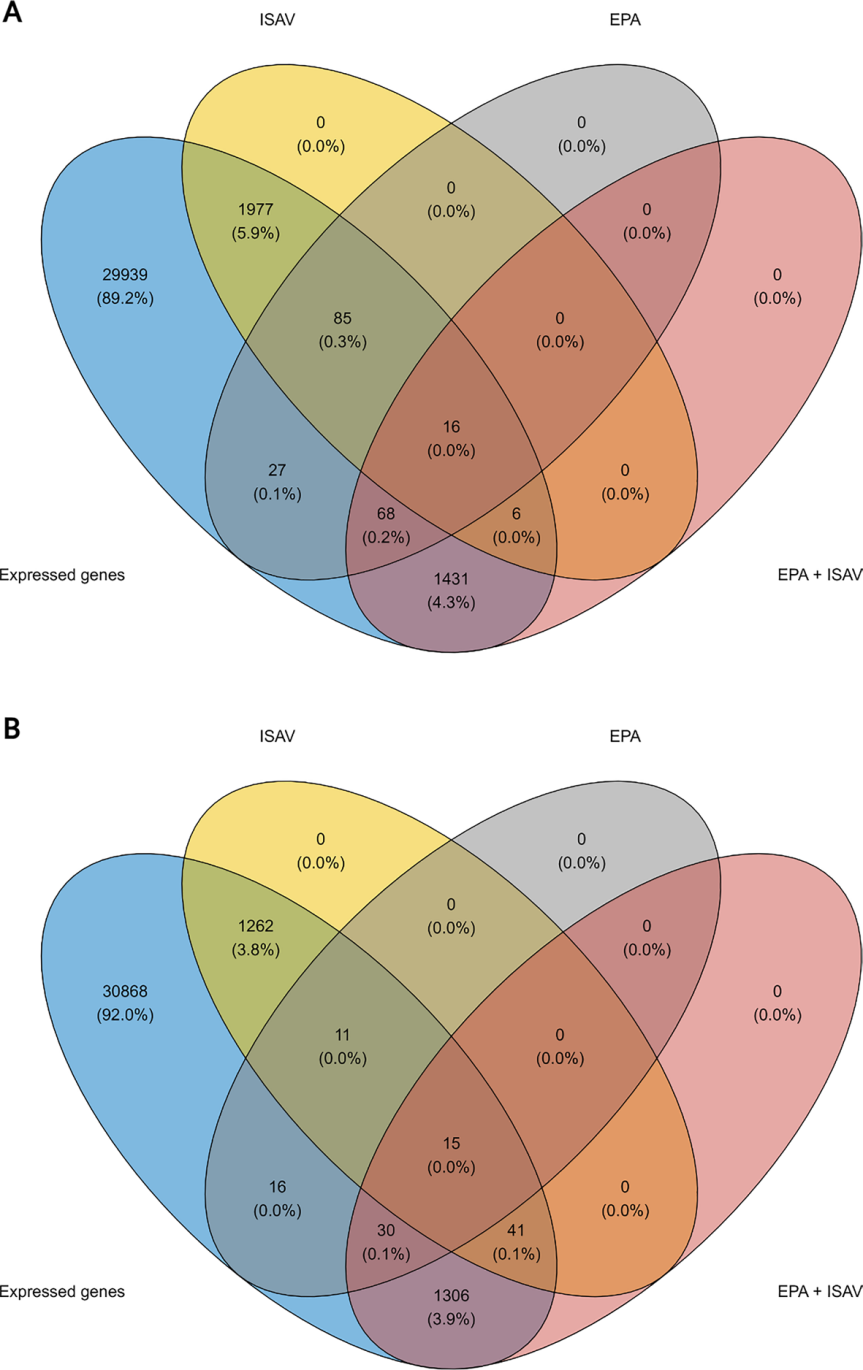
Venn diagrams showing overlap of significant DEGs in the various experimental groups.** A**: Upregulated genes.** B**: Downregulated genes

The overlap of transcripts between DEGs induced by ISAV alone and DEGs affected by the level of EPA was limited (Fig. 6). Only 120 of 3421 transcripts changed by viral infection were modulated by the cellular levels of EPA, suggesting that alternative signaling pathways were activated with increasing levels of the fatty acid.

**Fig. 7:**
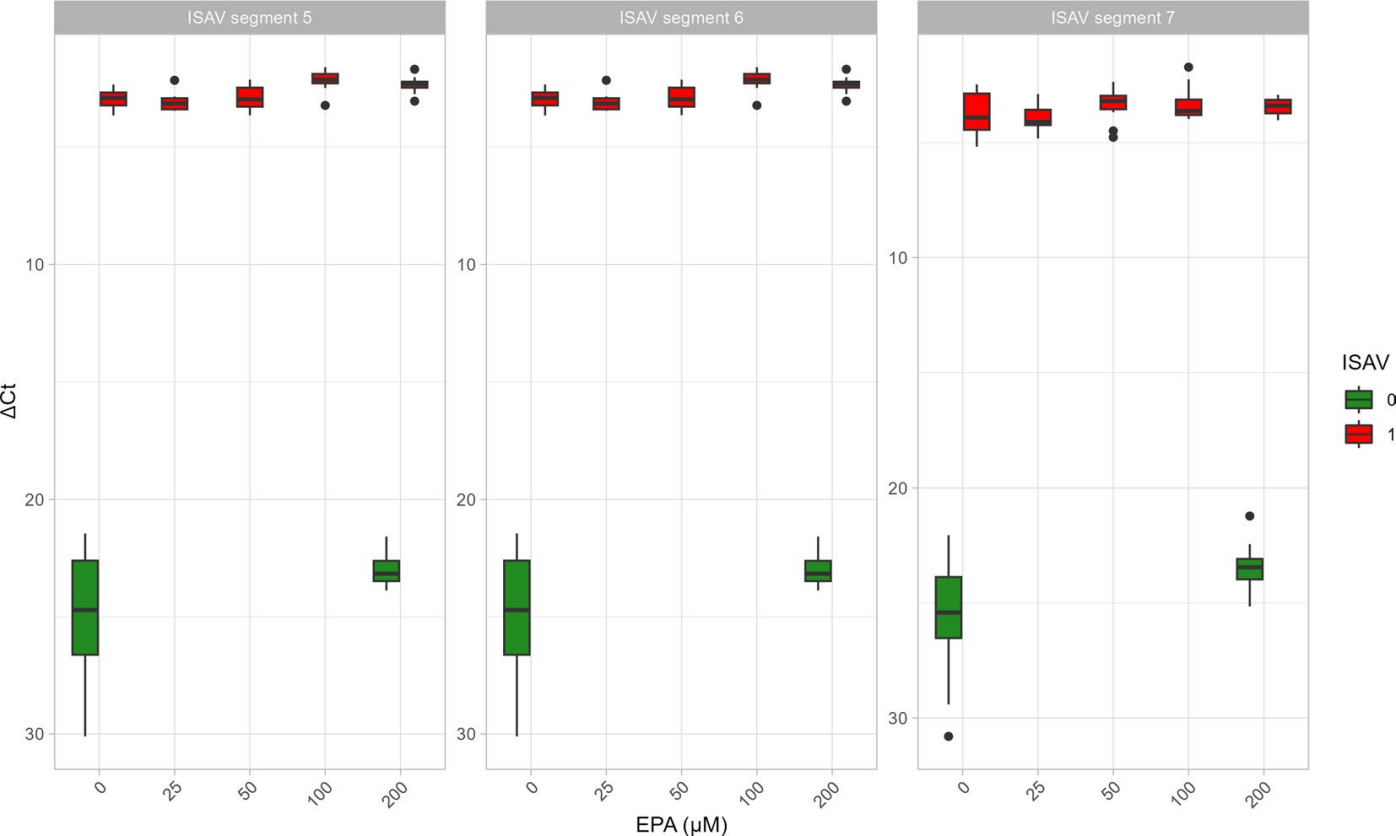
qPCR analysis of three ISAV gene segments in infected ASK cell cultures (red bars) or noninfected cells (green bars) at various EPA levels at 48 h after infection (*n* = 6)

When analyzing the levels of viral transcripts (ISAV segments 5, 6, and 7) at 48 h p.i. we did not observe a significant effect of EPA levels on viral replication (Fig. 7).

**Fig. 8:**
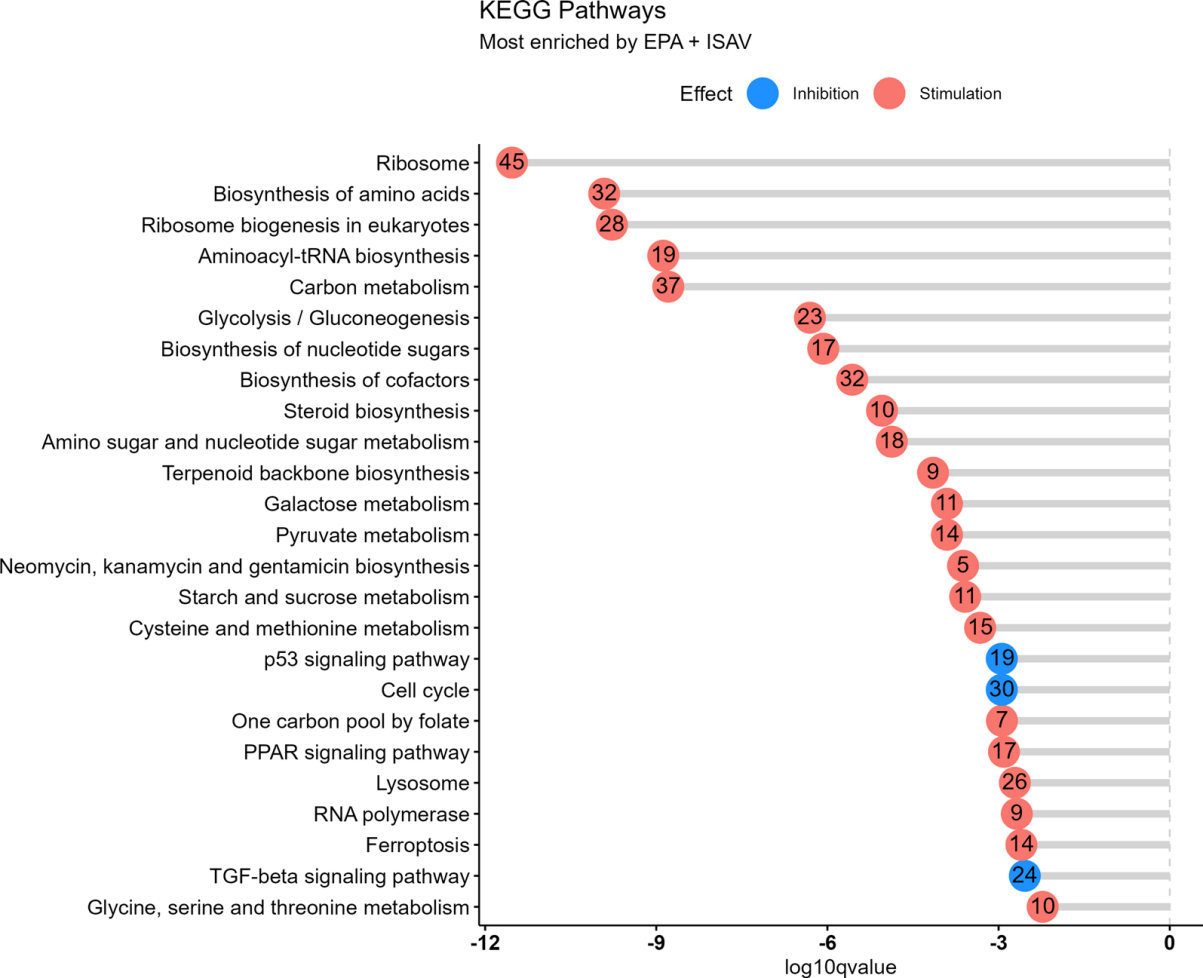
KEGG pathway enrichment analysis (biological processes) of gene sets significantly affected by EPA levels in ISAV infected ASK cells. Count represents number of transcripts in gene set. Color represents direction of EPA effect (blue = inhibition, red = stimulation)

KEGG pathway enrichment analysis with gene sets that were affected by EPA revealed that processes related to protein synthesis, amino acid, RNA metabolism, PPAR pathway, fatty metabolism, and ferroptosis were enriched with transcripts stimulated by EPA (Fig. 8). Cell cycle, p53 pathway, and TGF-beta signaling pathways were enriched with transcripts inhibited by EPA.

**Fig. 9:**
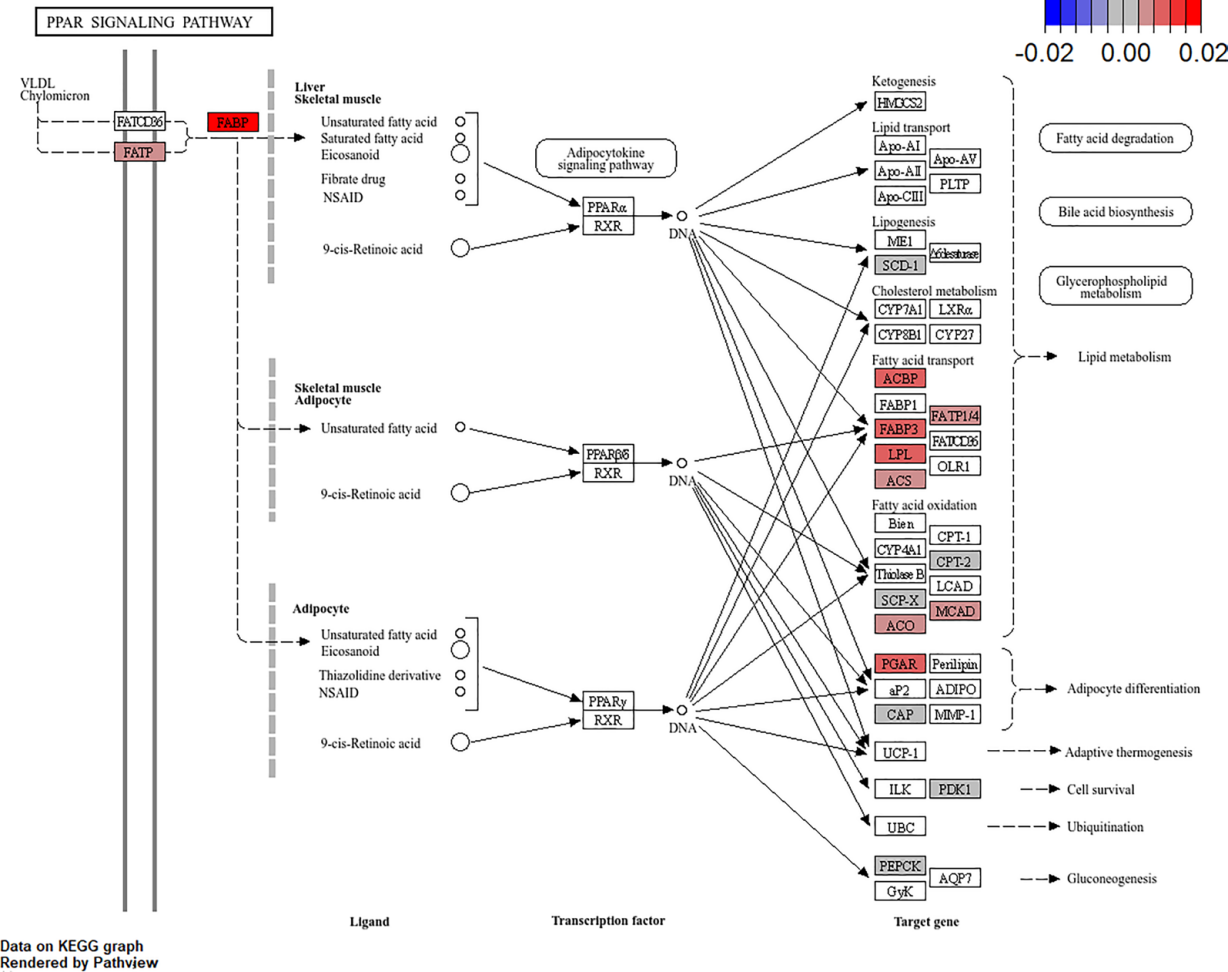
Effect of EPA on transcripts in the PPAR pathway during ISAV infection in ASK cells

Taking a closer look at the affected transcripts in the PPAR pathway revealed that all transcripts connected to this pathway were upregulated by EPA. Target genes for *PPAR-alpha, -delta, and -gamma* were affected by EPA in ISAV infected cells (Fig. 9).

**Fig. 10:**
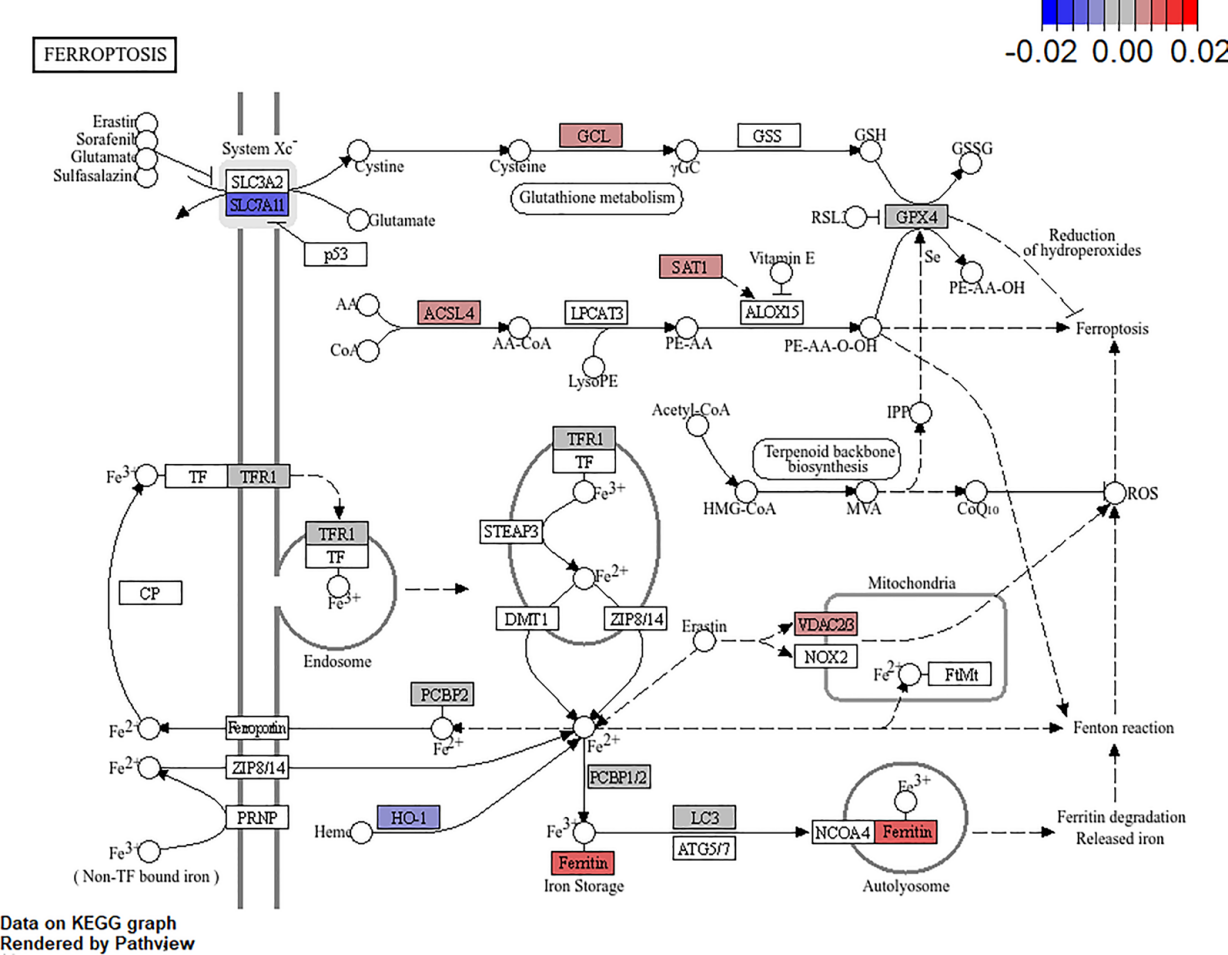
Effect of EPA on transcripts in the ferroptosis pathway during ISAV infection in ASK cells

Enrichment of transcripts in the ferroptosis pathway was another interesting feature of transcriptional changes observed with increasing EPA levels not seen in control cells infected with ISAV. Ferroptosis is a regulated iron dependent cell death pathway characterized by reduced antioxidant capacity, accumulation of lipid peroxides, and reactive oxygen species [43]. High levels of ferritin combined with reduced levels of the system Xc^−^ subunit *SLC7A11* (transporter for glutathione precursor cysteine) and glutathione peroxidase 4 (*GPX4*) was observed in EPA treated cells infected with ISAV, which may trigger activation of this pathway (Fig. 10).

## Discussion

Recent developments in the understanding of the interplay between dietary fatty acids and the immune responses at the cellular and organismal levels [40, 72, 73] requires further investigations of these relationships in farmed aquatic animals where feed is one of the main material factors [32, 74]. Previous reports suggest that salmon feeds with higher levels of EPA may confer a protective effect against viral [75, 76] and bacterial infections [77]. However, the minimal dietary requirements for EPA in salmon feed are not firmly established and may be dependent on developmental stage and other environmental factors [78–80]. Some reports conclude that EPA is not essential for normal health and growth of Atlantic salmon [39]. Nevertheless, that study was a short-term study (14 weeks) and used small fish of approximately 53 g at the beginning and concluded when fish reached approximately 200 g at the end of the feeding trial. Further, prior to the experiment a commercial diet was used. Commercial diets at this life stage are typically rich in n-3 VLC-PUFAs and this may have provided enough n-3 PUFAs to be sustained by the fish during the short feeding trial.

In this report we have studied the transcriptomic responses to ISAV in salmon cells under various cellular levels of EPA (from 2 to 21% of total fatty acids). We first confirmed the infection model by qPCR of established interferon regulated transcripts and viral genomic segments and found that the cells were robustly infected. RNA-seq analysis also confirmed the transcriptomic changes resulting from this infection in cells cultured in standard medium [54]. The sampling time point (48 h p.i.) was based on previous kinetic studies [54], capturing the innate transcriptional response in these cells. However, this sample was probably too early to capture effects of EPA on viral replication, as this normally takes about 72 h to develop quantifiable levels [81]. Results from pathway enrichment analysis were similar but not identical to a comparable study using the interferon inducer poly I:C instead of virus as the immunological stimuli [82]. The effects of poly I:C in combination with elevated levels of EPA were more restricted in the sense that multiple pathways like ECM-receptor interaction, autophagy, apelin, and VEGF-signaling were suppressed. Incubation with the highest concentration of EPA alone without any additional inflammatory stimuli had limited effects on the transcriptional profile of the cells and did not reveal significantly enriched GO or KEGG terms. This suggests that the highest concentration (200 µM) was well tolerated by the cells. However, combined with ISAV infection as the inflammatory signal, robust transcriptional changes involving multiple metabolic pathways were observed at higher levels of EPA. In addition to more general pathways like ribosome, carbohydrate, amino acid, and fatty acid metabolism, pathways enriched by EPA were ferroptosis, PPAR signaling, and lysosomal pathways. Transcripts downregulated by EPA were involved in cell cycle p53- and TGF-beta signaling (Fig. 7). These observations are in line with results obtained using poly I:C as inflammatory stimuli [82]. Although originally described as regulators of lipid metabolism, PPARs are now also recognized for their role in controlling inflammation induced by lipopolysaccharides [83] or via inhibition of interferon production [84]. In our experimental in vitro model, higher levels of EPA may therefore attenuate inflammatory responses to viral infection via activation of PPAR pathways, similar to effects observed with other viruses [85]. In dietary studies of Atlantic salmon combined with outbreaks of viral disease, a protective effect of higher PUFA levels was observed [86].

Ferroptosis is a mechanism of controlled cell death characterized by increased cellular Fe^2+^ concentration, iron-dependent oxidation of unsaturated membrane fatty acids, and mitochondrial contraction. This cell death can be experimentally induced by inhibition of cysteine uptake (precursor for the antioxidant glutathione) via the amino acid transporter system X_c_^−^, inactivation/reduction of glutathione peroxidase 4 (*GPX4*), depletion of coenzyme Q10, or lipid peroxidation due to PUFA overload [87] (Fig. 10). The observed downregulation of system X_c_^−^ combined with high levels of ferritin (and hence stored iron) in ISAV infected ASK cells may explain the triggering of ferroptosis observed in our study. How ferroptosis contributes to physiological homeostasis is not completely understood but it may play a role in tumor suppression [88], immunity [89], and development [90]. Recent studies suggest that ferroptosis may limit viral replication and pathogenesis [91] and be a part of the host innate immune response limiting viral spread [92]. The role of ferroptosis during ISAV infection under high levels of PUFAs like EPA observed here needs confirmation by biochemical assays of iron and peroxidation products in addition to analyzing the effects of ferroptosis inhibitors and activators on viral replication. In reviewing the experience with PUFA supplements on murine models of infection the jury is still out on the main effects. Several reports have documented reduced survival of bacteria infected animals on dietary fish oils [93, 94], most probably due to their immunosuppressive effects. However, other studies suggested increased resistance to bacterial infection by PUFA supplementation duet to enhanced cytokine production [95]. Murine models of viral infection also display contrasting effects of PUFAs on disease outcome [96, 97]. This dichotomy demonstrates the fine-tuned response by the immune system tailored to the invading pathogen and that there is no “one size fits all” explanation for the immunomodulatory roles of PUFAs [98]. Given the important role and high levels of PUFAs in ISAVs most important host, farmed Atlantic salmon, further studies are therefore needed to optimize the levels of these feed ingredients to support disease resistance to this virus and other pathogens.

## Conclusion

The interplay between lipid metabolism and immunity is receiving increased attention and constitutes a major part of immunometabolism. In this study, we have shown that various cellular levels of EPA in Atlantic salmon cells affect the regulation of multiple transcripts involved in innate immune responses to viral infection. At high levels of EPA, viral infection may precipitate regulated cell death pathways like ferroptosis due to increased oxidative stress. This supports previous studies using other viruses [91] and encourages further investigations on the interplay between metabolism and immunity in this species.

## Supplementary Information

Supplementary file 1: This file contains results from the fatty acid analysis, the exploratory plots of the RNA-seq data and a picture of virus infected cells.

Supplementary file 2: Tables with significantdifferentially expressed genesin the various experimental groups.

## Data Availability

Raw data from this project is available from the SRA archive (Bioproject ID: PRJNA1113821).

## Abbreviations

ACP: Acyl carrier protein
ASK: Atlantic salmon kidney
BH: Benjamini-Hochberg
Ct: Cycle threshold
DEG: Differentially expressed genes
DHA: Docosahexaenoic acid
ECM: Extracellular matrix
EPA: Eicosapentaenoic acid
FADS2: Fatty acid desaturase 2
GO: Gene ontology
GPR120: G-protein coupled receptor 120
GPX4: Glutathione peroxidase 4
IFIH1: Interferon induced with helicase C domain 1
IFNa: Interferon alpha-1
ISAV: Infectious salmon anemia virus
ISG15: Interferon-stimulated gene 15
KEGG: Kyoto Encyclopedia of Genes and Genomes
MAVS: Mitochondrial antiviral-signaling protein
MOI: Multiplicity of infection
Mx1: Interferon-induced GTP-binding protein Mx1
OLAH: Oleoyl-ACP hydrolase
PCA: Principal component analysis
Poly I:C: Polyinosinic-polycytidylic acid
PPAR: Peroxisome proliferator activated receptor
p.i.: Post-infection
STING: Stimulator of interferon genes
TGF: Transforming growth factor
VEGF: Vascular endothelial growth factor
VLC-PUFA: Very-long-chain polyunsaturated fatty acids

## References

[1] Thorud K, Djupvik H. Infectious anaemia in Atlantic salmon (Salmo salar L). Bull Eur Assoc Fish Pathol. 1988;8(5):109–11.

[2] Aamelfot M, Dale OB, Falk K. Infectious salmon anaemia - pathogenesis and tropism. J Fish Dis. 2014;37(4):291–307. 24475971 10.1111/jfd.12225

[3] Godoy MG, Kibenge MJ, Suarez R, Lazo E, Heisinger A, Aguinaga J, et al. Infectious salmon anaemia virus (ISAV) in Chilean Atlantic salmon (Salmo salar) aquaculture: emergence of low pathogenic ISAV-HPR0 and re-emergence of virulent ISAV-HPR∆: HPR3 and HPR14. virol J. 2013;10(1):344. 24268071 10.1186/1743-422X-10-344 PMC4222741

[4] Hastein T, Hill BJ, Winton JR. Successful aquatic animal disease emergency programmes. Rev Sci Tech. 1999;18(1):214–27. 10190216 10.20506/rst.18.1.1161

[5] Dhar AK, Manna SK, Thomas Allnutt FC. Viral vaccines for farmed finfish. Virusdisease. 2014;25(1):1–17. 24426306 10.1007/s13337-013-0186-4 PMC3889245

[6] Knipe DM, Howley PM. Fields virology. Philadelphia, PA: Wolters Kluwer/Lippincott Williams & Wilkins Health; 2013.

[7] Aamelfot M, Dale OB, Weli SC, Koppang EO, Falk K. Expression of the infectious salmon anemia virus receptor on atlantic salmon endothelial cells correlates with the cell tropism of the virus. J Virol. 2012;86(19):10571–8. 22811536 10.1128/JVI.00047-12 PMC3457268

[8] Weli S, Aamelfot M, Dale O, Koppang E, Falk K. Infectious salmon anaemia virus infection of Atlantic salmon gill epithelial cells. Virol J. 2013;10(1):5. 23282149 10.1186/1743-422X-10-5 PMC3560113

[9] Eliassen TM, Frøystad MK, Dannevig BH, Jankowska M, Brech A, Falk K, et al. Initial events in infectious salmon anemia virus infection: evidence for the requirement of a low-pH step. J Virol. 2000;74(1):218–27. 10590109 10.1128/jvi.74.1.218-227.2000 PMC111531

[10] Hellebø A, Vilas U, Falk K, Vlasak R. Infectious Salmon Anemia Virus Specifically Binds to and Hydrolyzes 4-O-Acetylated Sialic Acids. J Virol. 2004;78(6):3055–62. 14990724 10.1128/JVI.78.6.3055-3062.2004 PMC353765

[11] Mjaaland S, Rimstad E, Falk K, Dannevig BH. Genomic characterization of the virus causing infectious salmon anemia in Atlantic salmon (Salmo salar L): an orthomyxo-like virus in a teleost. J Virol. 1997;71(10):7681–6. 9311851 10.1128/jvi.71.10.7681-7686.1997 PMC192118

[12] Rimstad E, Mjaaland S. Infectious salmon anaemia virus. APMIS: Acta Pathologica, Microbiologica, Et Immunologica Scandinavica. 2002;110(4):273–82. 12076262 10.1034/j.1600-0463.2002.100401.x

[13] Jensen I, Albuquerque A, Sommer A, Robertsen B. Effect of poly I: C on the expression of Mx proteins and resistance against infection by infectious salmon anaemia virus in Atlantic salmon. Fish Shellfish Immunol. 2002;13(4):311–26. 12443013 10.1006/fsim.2001.0406

[14] Djordjevic B, Skugor S, Jorgensen SM, Overland M, Mydland LT, Krasnov A. Modulation of splenic immune responses to bacterial lipopolysaccharide in rainbow trout (Oncorhynchus mykiss) fed lentinan, a beta-glucan from mushroom Lentinula edodes. Fish Shellfish Immunol. 2009;26(2):201–9. 19010422 10.1016/j.fsi.2008.10.012

[15] LeBlanc F, Arseneau JR, Leadbeater S, Glebe B, Laflamme M, Gagne N. Transcriptional response of Atlantic salmon (Salmo salar) after primary versus secondary exposure to infectious salmon anemia virus (ISAV). Mol Immunol. 2012;51(2):197–209. 22475434 10.1016/j.molimm.2012.03.021

[16] Kibenge MT, Opazo B, Rojas AH, Kibenge FS. Serological evidence of infectious salmon anaemia virus (ISAV) infection in farmed fishes, using an indirect enzyme-linked immunosorbent assay (ELISA). Dis Aquat Org. 2002;51(1):1–11. 10.3354/dao051001 12240966

[17] Hetland DL, Jorgensen SM, Skjodt K, Dale OB, Falk K, Xu C, et al. In situ localisation of major histocompatibility complex class I and class II and CD8 positive cells in infectious salmon anaemia virus (ISAV)-infected Atlantic salmon. Fish Shellfish Immunol. 2010;28(1):30–9. 19766193 10.1016/j.fsi.2009.09.011

[18] Lauscher A, Krossoy B, Frost P, Grove S, Konig M, Bohlin J, et al. Immune responses in Atlantic salmon (Salmo salar) following protective vaccination against Infectious salmon anemia (ISA) and subsequent ISA virus infection. Vaccine. 2011. 10.1016/j.vaccine.2011.04.074. 21554914 10.1016/j.vaccine.2011.04.074

[19] Kossack C, Fuentes N, Maisey K. In silico prediction of B and T cell epitopes of infectious salmon anemia virus proteins and molecular modeling of T cell epitopes to salmon major histocompatibility complex (MHC) class I. Fish Shellfish Immunol. 2022;128:335–47. 35963560 10.1016/j.fsi.2022.08.002

[20] Toro-Ascuy D, Cardenas M, Vasquez-Martinez Y, Cortez-San MM. Rescue of infectious salmon anemia virus (ISAV) from cloned cDNA. Methods Mol Biol. 2024;2733:87–99. 38064028 10.1007/978-1-0716-3533-9_6

[21] Hwang D. Essential fatty acids and immune response. Faseb J. 1989;3(9):2052–61. 2501132 10.1096/fasebj.3.9.2501132

[22] Dustin LB, Shea CM, Soberman RJ, Lu CY. Docosahexaenoic acid, a constituent of rodent fetal serum and fish oil diets, inhibits acquisition of macrophage tumoricidal function. J Immunol. 1990;144(12):4888–97. 2141046

[23] Waagbo R. The impact of nutritional factors on the immune system in Atlantic salmon, Salmo salar L: a review. Aquaculture Res. 1994;25(2):175–97.

[24] Calder PC. n-3 polyunsaturated fatty acids and cytokine production in health and disease. Ann Nutr Metab. 1997;41(4):203–34. 9363294 10.1159/000177997

[25] March BE. Essential fatty acids in fish physiology. Can J Physiol Pharmacol. 1993;71:684–9. 8313232 10.1139/y93-102

[26] Velotti F, Costantini L, Merendino N. Omega-3 polyunsaturated fatty acids (n-3 PUFAs) for immunomodulation in COVID-19 related acute respiratory distress syndrome (ARDS). J Clin Med. 2022;12(1):304. 36615103 10.3390/jcm12010304 PMC9820910

[27] Mori TA, Beilin LJ. Omega-3 fatty acids and inflammation. Curr Atheroscler Rep. 2004;6(6):461–7. 15485592 10.1007/s11883-004-0087-5

[28] Miles EA, Calder PC. Influence of marine n-3 polyunsaturated fatty acids on immune function and a systematic review of their effects on clinical outcomes in rheumatoid arthritis. Br J Nutr. 2012;107(Suppl 2):S171–84. 22591891 10.1017/S0007114512001560

[29] Mebarek S, Ermak N, Benzaria A, Vicca S, Dubois M, Nemoz G, et al. Effects of increasing docosahexaenoic acid intake in human healthy volunteers on lymphocyte activation and monocyte apoptosis. Br J Nutr. 2009;101(6):852–8. 18710607 10.1017/S0007114508051520 PMC2685418

[30] Gallo CG, Fiorino S, Posabella G, Antonacci D, Tropeano A, Pausini E, et al. The function of specialized pro-resolving endogenous lipid mediators, vitamins, and other micronutrients in the control of the inflammatory processes: Possible role in patients with SARS-CoV-2 related infection. Prostaglandins Other Lipid Mediat. 2022;159:106619. 35032665 10.1016/j.prostaglandins.2022.106619 PMC8752446

[31] Ayres JS. Immunometabolism of infections. Nat Rev Immunol. 2020;20(2):79–80. 31892735 10.1038/s41577-019-0266-9

[32] Aas TS, Åsgård T, Ytrestøyl T. Utilization of feed resources in the production of Atlantic salmon (Salmo salar) in Norway: an update for 2020. Aquaculture Reports. 2022;26:101316.

[33] Ruyter RosjO, Einen T. Essential fatty acids in Atlantic salmon: effects of increasing dietary doses of n-6 and n-3 fatty acids on growth, survival and fatty acid composition of liver, blood and carcass. Aquac Nutr. 2000;6(2):119–27.

[34] Bell JG, Sargent JR, Raynard RS. Effects of increasing dietary linoleic acid on phospholipid fatty acid composition and eicosanoid production in leucocytes and gill cells of Atlantic salmon (Salmo salar). Prostaglandins Leukot Essent Fatty Acids. 1992;45(3):197–206. 1589446 10.1016/0952-3278(92)90113-w

[35] Bell JG, Tocher DR, Farndale BM, Cox DI, McKinney RW, Sargent JR. The effect of dietary lipid on polyunsaturated fatty acid metabolism in Atlantic salmon (*Salmo salar*) undergoing Parr- Smolt transformation. Lipids. 1997;32(5):515–25. 9168458 10.1007/s11745-997-0066-4

[36] Bell JG, Ashton I, Secombes CJ, Weitzel BR, Dick JR, Sargent JR. Dietary lipid affects phospholipid fatty acid compositions, eicosanoid production and immune function in Atlantic salmon (Salmo salar). Prostaglandins Leukot Essent Fatty Acids. 1996;54:173–82. 8860104 10.1016/s0952-3278(96)90013-7

[37] Holen E, Araujo P, Sissener NH, Rosenlund G, Waagbø R. A comparative study: Difference in omega-6, omega-3 balance and saturated fat in diets for Atlantic salmon (Salmo salar) affect immune-, fat metabolism-, oxidative and apoptotic-gene expression, and eicosanoid secretion in head kidney leukocytes. Fish Shellfish Immunol. 2018;72:57. 29080687 10.1016/j.fsi.2017.10.040

[38] Andresen AMS, Lutfi E, Ruyter B, Berge G, Gjoen T. Interaction between dietary fatty acids and genotype on immune response in Atlantic salmon (Salmo salar) after vaccination: A transcriptome study. PLoS ONE. 2019;14(7):e0219625. 31365530 10.1371/journal.pone.0219625 PMC6668776

[39] Emery JA, Norambuena F, Trushenski J, Turchini GM. Uncoupling EPA and DHA in fish nutrition: dietary demand is limited in atlantic salmon and effectively met by DHA alone. Lipids. 2016;51(4):399–412. 26965251 10.1007/s11745-016-4136-y

[40] Vila IK, Chamma H, Steer A, Saccas M, Taffoni C, Turtoi E, et al. STING orchestrates the crosstalk between polyunsaturated fatty acid metabolism and inflammatory responses. Cell Metab. 2022;34(1):125–39. 34986331 10.1016/j.cmet.2021.12.007 PMC8733004

[41] Zhang W, Wang G, Xu ZG, Tu H, Hu F, Dai J, et al. Lactate is a natural suppressor of RLR signaling by targeting MAVS. Cell. 2019;178(1):176–89. 31155231 10.1016/j.cell.2019.05.003 PMC6625351

[42] Jia X, Crawford JC, Gebregzabher D, Monson EA, Mettelman RC, Wan Y, et al. High expression of oleoyl-ACP hydrolase underpins life-threatening respiratory viral diseases. Cell. 2024;187(17):4586-604.e20. 39137778 10.1016/j.cell.2024.07.026

[43] Dixon SJ, Lemberg KM, Lamprecht MR, Skouta R, Zaitsev EM, Gleason CE, et al. Ferroptosis: an iron-dependent form of nonapoptotic cell death. Cell. 2012;149(5):1060–72. 22632970 10.1016/j.cell.2012.03.042 PMC3367386

[44] Zhao X, Zhang Y, Luo B. Ferroptosis, from the virus point of view: opportunities and challenges. Crit Rev Microbiol. 2024. 10.1080/1040841X.2024.2340643. 38588443 10.1080/1040841X.2024.2340643

[45] Calder PC. The relationship between the fatty acid composition of immune cells and their function. Prostaglandins Leukot Essent Fatty Acids. 2008;79(3–5):101–8. 18951005 10.1016/j.plefa.2008.09.016

[46] Serhan CN, Chiang N, Van Dyke TE. Resolving inflammation: dual anti-inflammatory and pro-resolution lipid mediators. Nat Rev Immunol. 2008;8(5):349–61. 18437155 10.1038/nri2294 PMC2744593

[47] Motshwene PG, Moncrieffe MC, Grossmann JG, Kao C, Ayaluru M, Sandercock AM, et al. An oligomeric signaling platform formed by the Toll-like receptor signal transducers MyD88 and IRAK-4. J Biol Chem. 2009;284(37):25404–11. 19592493 10.1074/jbc.M109.022392 PMC2757241

[48] Hwang Keun Y, Choi YB. Modulation of mitochondrial antiviral signaling by human herpesvirus 8 interferon regulatory factor 1. J Virol. 2015;90(1):506–20. 26512076 10.1128/JVI.01903-15 PMC4702585

[49] Arnemo M, Kavaliauskis A, Andresen AMS, Bou M, Berge GM, Ruyter B, Gjoen T. Effects of dietary n-3 fatty acids on Toll-like receptor activation in primary leucocytes from Atlantic salmon (Salmo salar). Fish Physiol Biochem. 2017;43(4):1065–80. 28280951 10.1007/s10695-017-0353-4

[50] Gjøen T, Kleveland EJ, Moya-Falcón C, Frøystad MK, Vegusdal A, Hvattum E, et al. Effects of dietary thia fatty acids on lipid composition, morphology and macrophage function of Atlantic salmon ( Salmo salar L) kidney. Comp Biochem Physiol Part B: Biochem Molecular Biol. 2007;148(1):103–11. 10.1016/j.cbpb.2007.04.021 17572126

[51] Metochis CP, Spanos I, Auchinachie N, Crampton VO, Bell JG, Adams A, Thompson KD. The effects of increasing dietary levels of soy protein concentrate (SPC) on the immune responses and disease resistance (furunculosis) of vaccinated and non-vaccinated Atlantic salmon (Salmo salar L.) parr. Fish Shellfish Immunol. 2016;59:83–94. 27742588 10.1016/j.fsi.2016.10.016

[52] Caballero-Solares A, Hall JR, Xue X, Eslamloo K, Taylor RG, Parrish CC, Rise ML. The dietary replacement of marine ingredients by terrestrial animal and plant alternatives modulates the antiviral immune response of Atlantic salmon (Salmo salar). Fish Shellfish Immunol. 2017;64:24–38. 28242361 10.1016/j.fsi.2017.02.040

[53] Mjaaland S, Markussen T, Sindre H, Kjoglum S, Dannevig BH, Larsen S, Grimholt U. Susceptibility and immune responses following experimental infection of MHC compatible Atlantic salmon (Salmo salar L) with different infectious salmon anaemia virus isolates. Arch Virol. 2005;150(11):2195–216. 16012784 10.1007/s00705-005-0588-8

[54] Andresen AMS, Boudinot P, Gjoen T. Kinetics of transcriptional response against poly (I:C) and infectious salmon anemia virus (ISAV) in Atlantic salmon kidney (ASK) cell line. Dev Comp Immunol. 2020;110:103716. 32360383 10.1016/j.dci.2020.103716

[55] Folch J, Lees M, Stanley GHS. A simple method for the isolation and purification of total lipides from animal tissues. J Biol Chem. 1957;226(1):497–509. 13428781

[56] Mason ME, Waller GR. Dimethoxypropane induced transesterification of fats and oils in preparation of methyl esters for gas chromatographic analysis. Anal Chem. 1964;36(3):583–6.

[57] Hoshi M, Williams M, Kishimoto Y. Esterification of fatty acids at room temperature by chloroform-methanolic HCl–cupric acetate. J Lipid Res. 1973;14(5):599–601. 4729977

[58] Pfaffl MW. A new mathematical model for relative quantification in real-time RT–PCR. Nucleic Acids Res. 2001;29(9):e45. 11328886 10.1093/nar/29.9.e45 PMC55695

[59] Jørgensen SM, Kleveland EJ, Grimholt U, Gjøen T. Validation of reference genes for real-time polymerase chain reaction studies in Atlantic salmon. Mar Biotechnol. 2006;8(4):398–408. 10.1007/s10126-005-5164-4 16676145

[60] Jorgensen SM, Grimholt U, Gjoen T. Cloning and expression analysis of an Atlantic salmon (Salmo salar L) tapasin gene. Dev Comp Immunol. 2007;31(7):708–19. 17157378 10.1016/j.dci.2006.10.004

[61] Hansen TE, Jorgensen JB. Cloning and characterisation of p38 MAP kinase from Atlantic salmon A kinase important for regulating salmon TNF-2 and IL-1beta expression. Mol Immunol. 2007;44(12):3137–46. 17391766 10.1016/j.molimm.2007.02.006

[62] Schiotz BL, Baekkevold ES, Poulsen LC, Mjaaland S, Gjoen T. Analysis of host- and strain-dependent cell death responses during infectious salmon anemia virus infection in vitro. Virol J. 2009;6:91. 19566966 10.1186/1743-422X-6-91 PMC2715388

[63] Jorgensen SM, Lyng-Syvertsen B, Lukacs M, Grimholt U, Gjoen T. Expression of MHC class I pathway genes in response to infectious salmon anaemia virus in Atlantic salmon (Salmo salar L) cells. Fish Shellfish Immunol. 2006;21(5):548–60. 16772112 10.1016/j.fsi.2006.03.004

[64] Clouthier SC, Rector T, Brown NEC, Anderson ED. Genomic organization of infectious salmon anaemia virus. J Gen Virol. 2002;83(2):421–8. 11807235 10.1099/0022-1317-83-2-421

[65] Kim D, Paggi JM, Park C, Bennett C, Salzberg SL. Graph-based genome alignment and genotyping with HISAT2 and HISAT-genotype. Nat Biotechnol. 2019;37(8):907–15. 31375807 10.1038/s41587-019-0201-4 PMC7605509

[66] Pertea M, Pertea GM, Antonescu CM, Chang TC, Mendell JT, Salzberg SL. StringTie enables improved reconstruction of a transcriptome from RNA-seq reads. Nat Biotechnol. 2015;33(3):290–5. 25690850 10.1038/nbt.3122 PMC4643835

[67] Love MI, Huber W, Anders S. Moderated estimation of fold change and dispersion for RNA-seq data with DESeq2. Genome Biol. 2014;15(12):550. 25516281 10.1186/s13059-014-0550-8 PMC4302049

[68] Love MI, Anders S, Kim V, Huber W. RNA-Seq workflow: gene-level exploratory analysis and differential expression. F1000Research. 2015;4:1070. 26674615 10.12688/f1000research.7035.1 PMC4670015

[69] Benjamini Y, Hochberg Y. Controlling the false discovery rate: a practical and powerful approach to multiple testing. J Roy Stat Soc: Ser B Methodol. 1995;57(1):289–300.

[70] Yu G, Wang L-G, Han Y, He Q-Y. clusterprofiler: an R package for comparing biological themes among gene clusters. OMICS: J Integr Biol. 2012;16(5):284–7. 10.1089/omi.2011.0118 PMC3339379 22455463

[71] Luo W, Brouwer C. Pathview: an R/Bioconductor package for pathway-based data integration and visualization. Bioinformatics. 2013;29(14):1830–1. 23740750 10.1093/bioinformatics/btt285 PMC3702256

[72] Sularea VM, Sugrue JA, O’Farrelly C. Innate antiviral immunity and immunometabolism in hepatocytes. Curr Opin Immunol. 2023;80:102267. 36462263 10.1016/j.coi.2022.102267

[73] Liang D, Minikes AM, Jiang X. Ferroptosis at the intersection of lipid metabolism and cellular signaling. Mol Cell. 2022. 10.1016/j.molcel.2022.03.022. 35390277 10.1016/j.molcel.2022.03.022 PMC9233073

[74] Lutfi E, Berge GM, Bæverfjord G, Sigholt T, Bou M, Larsson T, et al. Increasing dietary levels of the n-3 long-chain PUFA, EPA and DHA, improves the growth, welfare, robustness and fillet quality of Atlantic salmon in sea cages. Br J Nutr. 2023;129(1):10–28. 35236527 10.1017/S0007114522000642 PMC9816656

[75] Martinez-Rubio L, Morais S, Evensen O, Wadsworth S, Ruohonen K, Vecino JL, et al. Functional feeds reduce heart inflammation and pathology in Atlantic Salmon (Salmo salar L) following experimental challenge with Atlantic salmon reovirus (ASRV). PLoS ONE. 2012;7(11):e40266. 23226193 10.1371/journal.pone.0040266 PMC3511526

[76] Martinez-Rubio L, Evensen O, Krasnov A, Jorgensen SM, Wadsworth S, Ruohonen K, et al. Effects of functional feeds on the lipid composition, transcriptomic responses and pathology in heart of Atlantic salmon (Salmo salar L) before and after experimental challenge with Piscine Myocarditis Virus (PMCV). BMC Genomics. 2014;15:462. 24919788 10.1186/1471-2164-15-462 PMC4079957

[77] Rorvik KA, Dehli A, Thomassen M, Ruyter B, Steien SH, Salte R. Synergistic effects of dietary iron and omega-3 fatty acid levels on survival of farmed Atlantic salmon, Salmo salar L, during natural outbreaks of furunculosis and cold water vibriosis. J Fish Dis. 2003;26(8):477–85. 14513972 10.1046/j.1365-2761.2003.00482.x

[78] Selvam C, Philip AJP, Lutfi E, Sigholt T, Norberg B, Baeverfjord G, et al. Long-term feeding of Atlantic salmon with varying levels of dietary EPA + DHA alters the mineral status but does not affect the stress responses after mechanical delousing stress. Br J Nutr. 2022;128(12):2291–307. 35156914 10.1017/S0007114522000514 PMC9723492

[79] Huyben D, Grobler T, Matthew C, Bou M, Ruyter B, Glencross B. Requirement for omega-3 long-chain polyunsaturated fatty acids by Atlantic salmon is relative to the dietary lipid level. Aquaculture. 2021;531:735805.

[80] Rosenlund G, Torstensen BE, Stubhaug I, Usman N, Sissener NH. Atlantic salmon require long-chain n-3 fatty acids for optimal growth throughout the seawater period. J Nutr Sci. 2016;5:e19. 27293556 10.1017/jns.2016.10 PMC4891698

[81] Svingerud T, Holand JK, Robertsen B. Infectious salmon anemia virus (ISAV) replication is transiently inhibited by Atlantic salmon type I interferon in cell culture. Virus Res. 2013;177(2):163–70. 23973914 10.1016/j.virusres.2013.08.004

[82] Gjøen T, Ruyter B, Østbye T-KK. Effects of eicosapentaneoic acid on innate immune responses in an Atlantic salmon kidney cell line in vitro. PLoS ONE. 2024. 10.1371/journal.pone.0302286. 38805503 10.1371/journal.pone.0302286 PMC11132502

[83] Heming M, Gran S, Jauch SL, Fischer-Riepe L, Russo A, Klotz L, et al. Peroxisome Proliferator-Activated Receptor-gamma Modulates the Response of Macrophages to Lipopolysaccharide and Glucocorticoids. Front Immunol. 2018;9:893. 29867927 10.3389/fimmu.2018.00893 PMC5949563

[84] Zhao W, Wang L, Zhang M, Wang P, Zhang L, Yuan C, Gao C. Peroxisome proliferator-activated receptor γ negatively regulates IFN-β production in Toll-like receptor (TLR) 3-and TLR4-stimulated macrophages by preventing interferon regulatory factor 3 binding to the IFN-β promoter. J Biol Chem. 2011;286(7):5519–28. 21148557 10.1074/jbc.M110.149823 PMC3037665

[85] Fantacuzzi MA-O, Amoroso RA-O, Ammazzalorso AA-O. PPAR Ligands Induce Antiviral Effects Targeting Perturbed Lipid Metabolism during SARS-CoV-2, HCV, and HCMV Infection. Biology. 2022. 10.3390/biology11010114. 35053112 10.3390/biology11010114 PMC8772958

[86] Lutfi E, Berge GM, Baeverfjord G, Sigholt T, Bou M, Larsson T, et al. Increasing dietary levels of the omega-3 long-chain polyunsaturated fatty acids, EPA and DHA, improves the growth, welfare, robustness, and fillet quality of Atlantic salmon in sea cages. Br J Nutr. 2022;129(1):1–48. 10.1017/S0007114522000642 PMC9816656 35236527

[87] Stockwell BR. Ferroptosis turns 10: Emerging mechanisms, physiological functions, and therapeutic applications. Cell. 2022;185(14):2401–21. 35803244 10.1016/j.cell.2022.06.003 PMC9273022

[88] Jiang L, Kon N, Li T, Wang SJ, Su T, Hibshoosh H, et al. Ferroptosis as a p53-mediated activity during tumour suppression. Nature. 2015;520(7545):57–62. 25799988 10.1038/nature14344 PMC4455927

[89] Yao Y, Chen Z, Zhang H, Chen C, Zeng M, Yunis J, et al. Selenium–GPX4 axis protects follicular helper T cells from ferroptosis. Nat Immunol. 2021;22(9):1127–39. 34413521 10.1038/s41590-021-00996-0

[90] Zheng H, Jiang L, Tsuduki T, Conrad M, Toyokuni S. Embryonal erythropoiesis and aging exploit ferroptosis. Redox Biol. 2021;48:102175. 34736120 10.1016/j.redox.2021.102175 PMC8577445

[91] Xu XQ, Xu T, Ji W, Wang C, Ren Y, Xiong X, et al. Herpes Simplex Virus 1-Induced Ferroptosis Contributes to Viral Encephalitis. MBio. 2023;14(1):e0237022. 36507835 10.1128/mbio.02370-22 PMC9973258

[92] Yamane D, Hayashi Y, Matsumoto M, Nakanishi H, Imagawa H, Kohara M, et al. FADS2-dependent fatty acid desaturation dictates cellular sensitivity to ferroptosis and permissiveness for hepatitis C virus replication. Cell Chem Biol. 2022;29(5):799-810.e4. 34520742 10.1016/j.chembiol.2021.07.022 PMC8913804

[93] De Pablo M, Puertollano MA, Galvez A, Ortega E, Gaforio J, Alvarez de Cienfuegos G. Determination of natural resistance of mice fed dietary lipids to experimental infection induced by Listeria monocytogenes. FEMS Immunol Medical Microbiol. 2000;27(2):127–33. 10.1111/j.1574-695X.2000.tb01422.x 10640607

[94] Fritsche KL, Shahbazian LM, Feng C, Berg JN. Dietary fish oil reduces survival and impairs bacterial clearance in C3H/Hen mice challenged with Listeria monocytogenes. Clin Sci (Lond). 1997;92(1):95–101. 9038598 10.1042/cs0920095

[95] Puertollano M, De Pablo M, Alvarez de Cienfuegos G. Immunomodulatory effects of dietary lipids alter host natural resistance of mice to Listeria monocytogenes infection. FEMS Immunol Med Microbiol. 2001;32(1):47–52. 11750222 10.1111/j.1574-695X.2001.tb00533.x

[96] Blok WL, Vogels MT, Curfs JH, Eling WM, Buurman WA, van der Meer JW. Dietary fish-oil supplementation in experimental gram-negative infection and in cerebral malaria in mice. J Infect Dis. 1992;165(5):898–903. 1569340 10.1093/infdis/165.5.898

[97] Blok WL, Katan MB, van der Meer JW. Modulation of inflammation and cytokine production by dietary (n-3) fatty acids. J Nutr. 1996;126(6):1515–33. 8648424 10.1093/jn/126.6.1515

[98] Anderson M, Fritsche KL. (n-3) Fatty acids and infectious disease resistance. J Nutr. 2002;132(12):3566–76. 12468590 10.1093/jn/132.12.3566

