# Supplementary material for: Effect of eicosapentaenoic acid on innate immune responses in Atlantic salmon cells infected with infectious salmon anemia virus
Source: Virol J. 2025 Jan 9;22:5. doi: 10.1186/s12985-024-02619-0 (PMC11715085; doi:10.1186/s12985-024-02619-0)
Supplement: Supplementary file 1 — This file contains results from the fatty acid analysis, the exploratory plots of the RNA-seq data and a picture of virus infected cells. [file 12985_2024_2619_MOESM1_ESM.pdf]

Supplementary file 1 - Effect of eicosapentaneoic acid on innate immune responses in Atlantic salmon cells infected with infectious salmon anemia virus

Ingrid Holmlund, Samira Ahmadi, Bente Ruyter, Tone-Kari Knutsdatter Østbye, Marta Bou and Tor Gjøen

December 10, 2024

This supplementary file provides sample information and exploratory plots for the manuscript "Effect of eicosapentaneoic acid on innate immune responses in Atlantic salmon cells infected with infectious salmon anemia virus". The data includes results from the fatty acid analysis, distributions of RNAseq count data, correlations between replicates, sample clustering, and an image of ASK cell culture infected with ISAV. The analysis has been performed using the R statistical programming language and R BioConductor packages (a complete list is given in the bottom of the document (Session info)). This supplementary file was generated using the knitr R package under Windows 10.

Figure S1

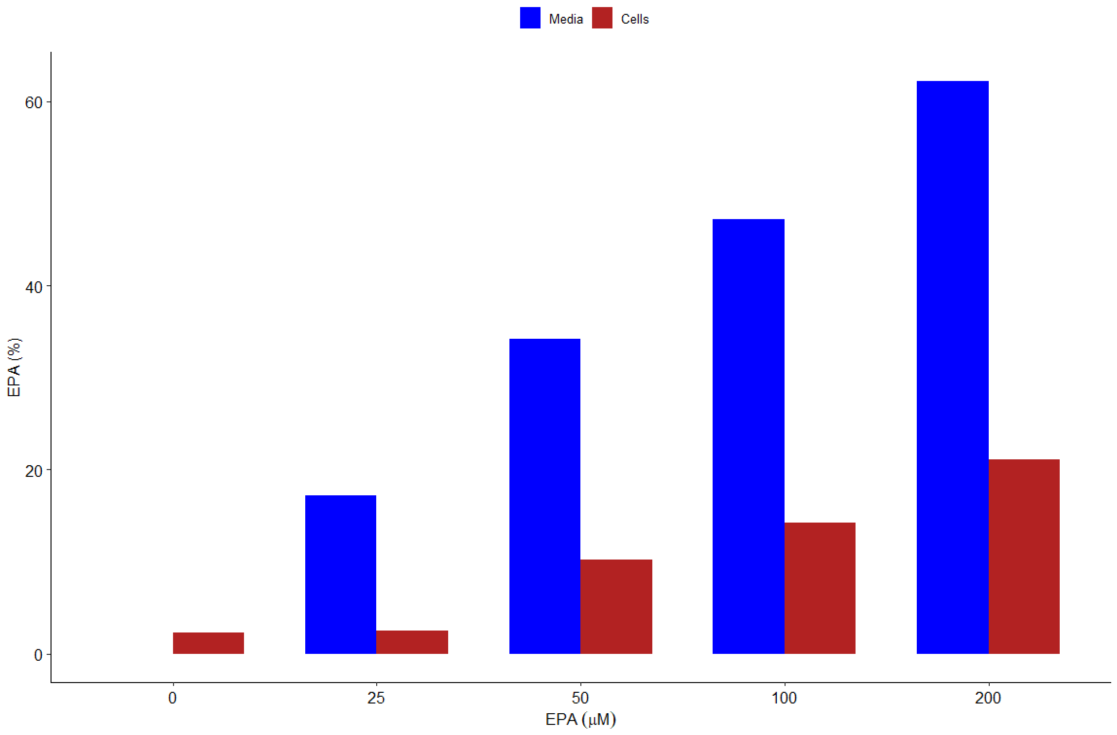

Bar plot display of EPA (%) in media and cells. The Folch method was used to extract the total lipids of cells and cell media after one week of cultivation. Increasing levels of EPA added to the media affect both the media and cell fatty acid composition.

Figure S2

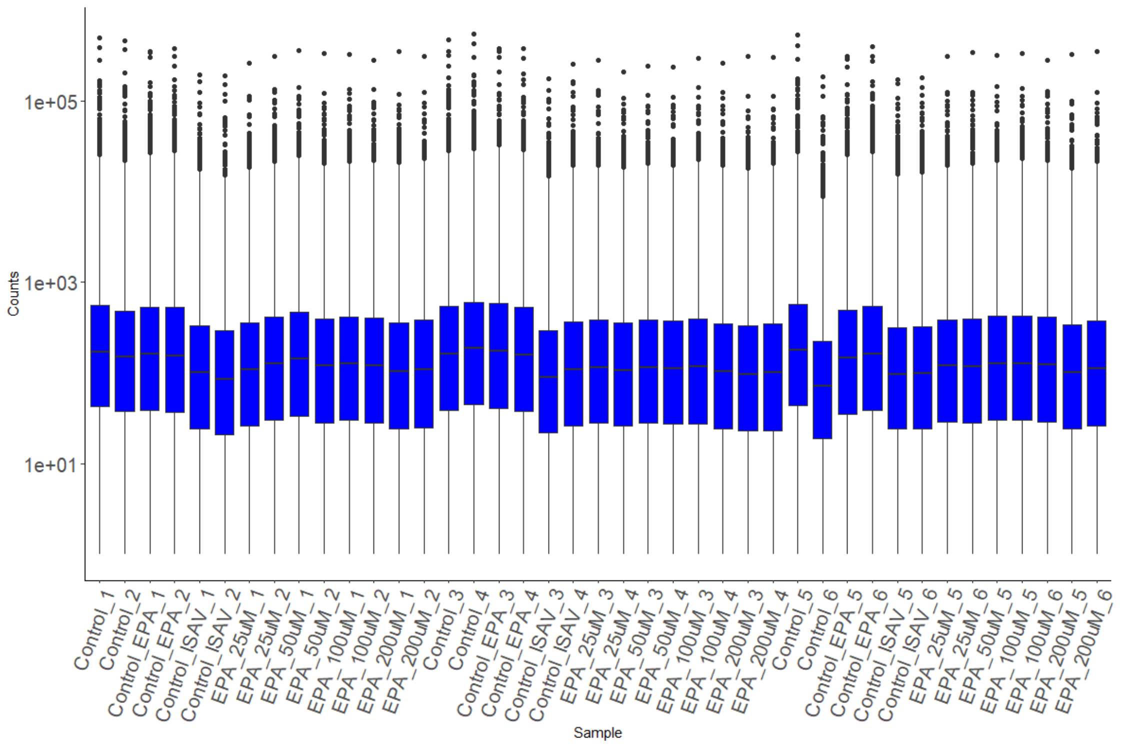

Boxplot display of the distribution of counts in each sample. Mean and variance were comparable in all samples.

Figure S3

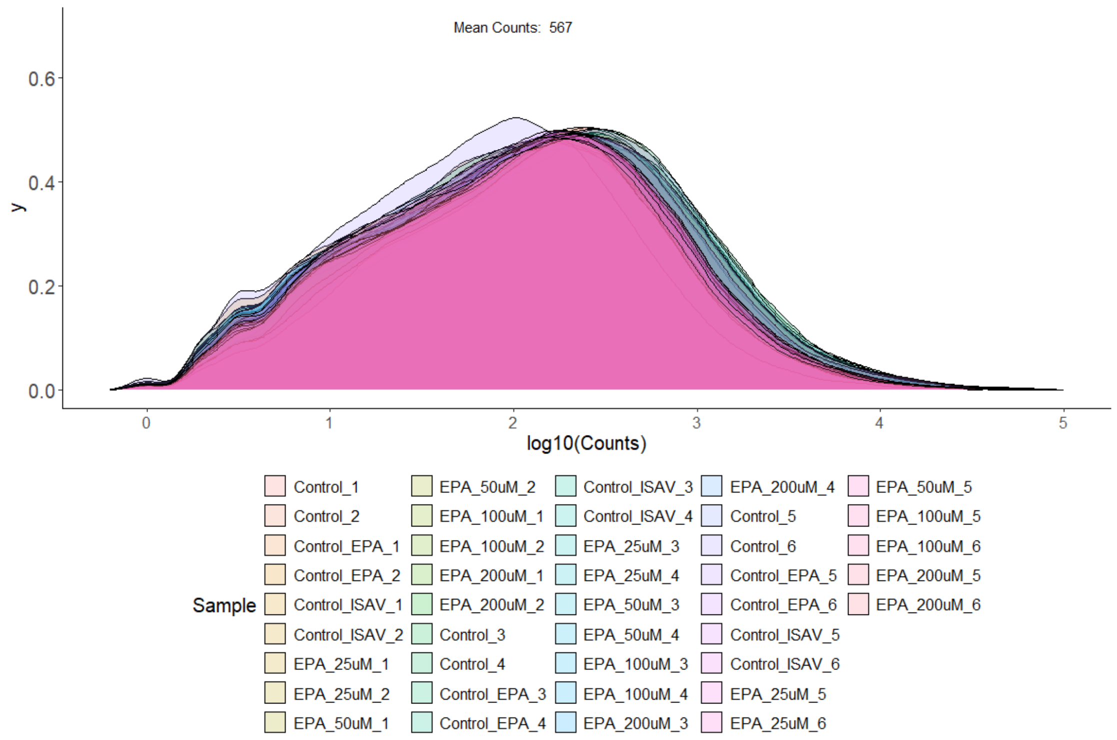

Density plot comparing the distribution of log10 raw counts in each sample.

Figure S4

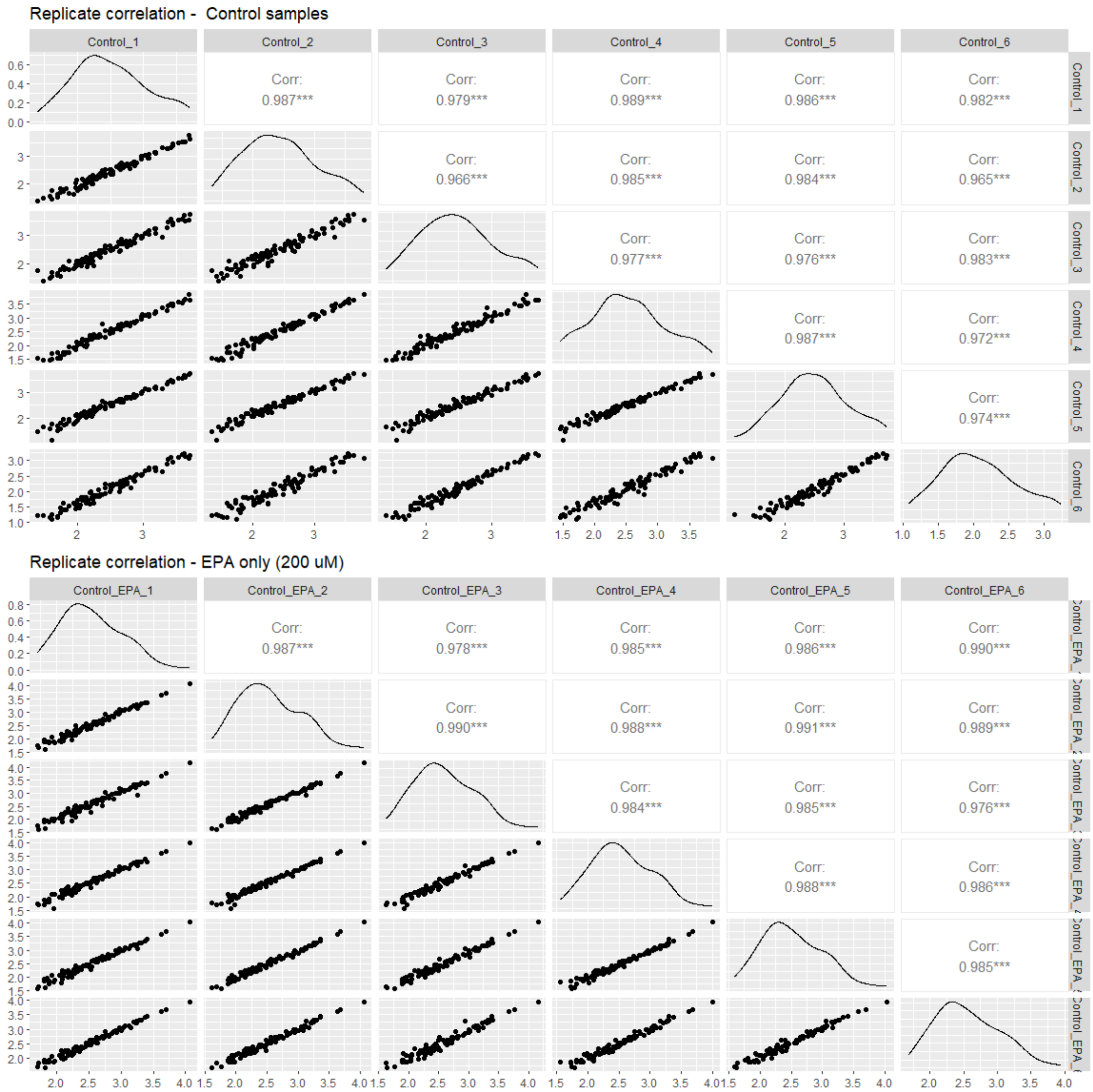

Replicate correlation - Control samples
Control_1
Control_2
Control_3
Control_4
Control_5
Control_6
Corr: 0.987***
Corr: 0.979***
Corr: 0.989***
Corr: 0.986***
Corr: 0.982***
Corr: 0.966***
Corr: 0.985***
Corr: 0.984***
Corr: 0.965***
Corr: 0.977***
Corr: 0.976***
Corr: 0.983***
Corr: 0.987***
Corr: 0.972***
Corr: 0.974***
Replicate correlation - EPA only (200 uM)
Control_EPA_1
Control_EPA_2
Control_EPA_3
Control_EPA_4
Control_EPA_5
Control_EPA_6
Corr: 0.987***
Corr: 0.978***
Corr: 0.985***
Corr: 0.986***
Corr: 0.990***
Corr: 0.990***
Corr: 0.988***
Corr: 0.991***
Corr: 0.989***
Corr: 0.984***
Corr: 0.985***
Corr: 0.976***
Corr: 0.988***
Corr: 0.986***
Corr: 0.985***

Replicate correlation - ISAV only

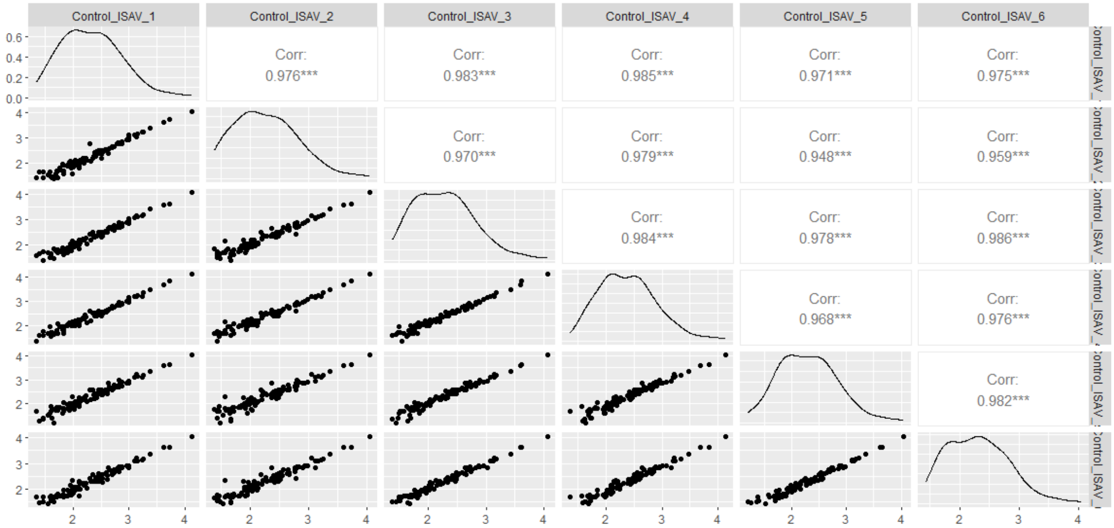

Replicate correlation - 25 uM EPA, ISAV

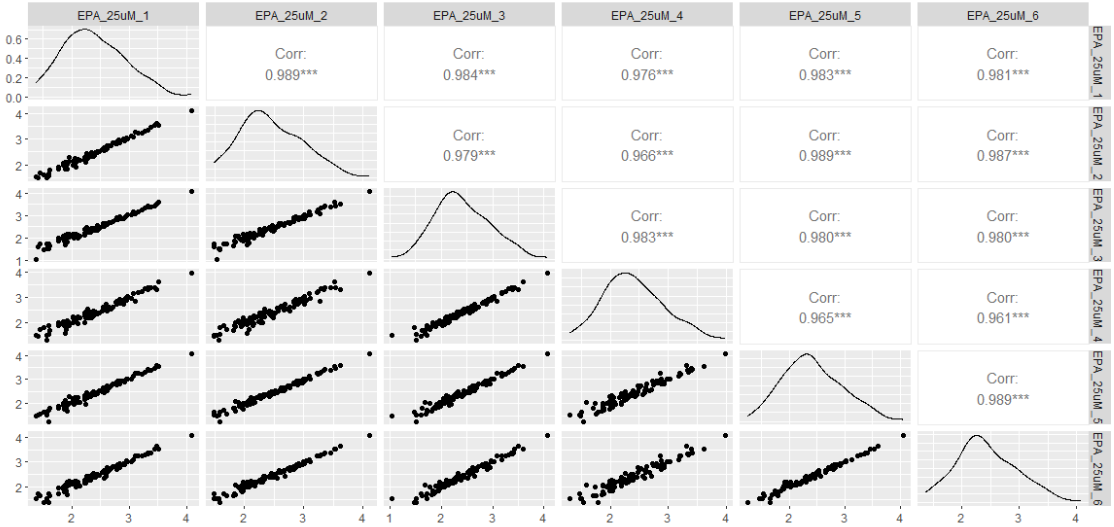

Replicate correlation - 50 uM EPA, ISAV

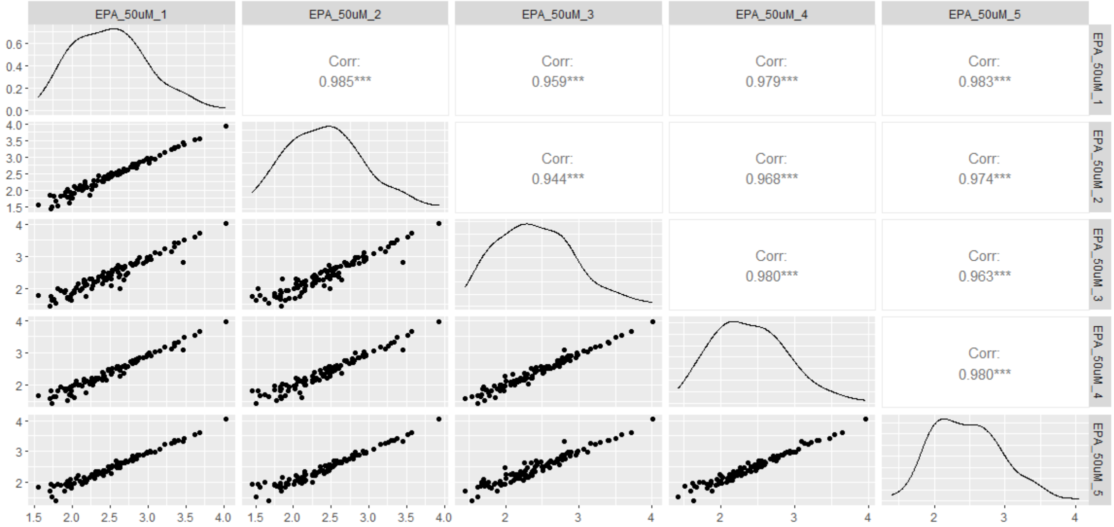

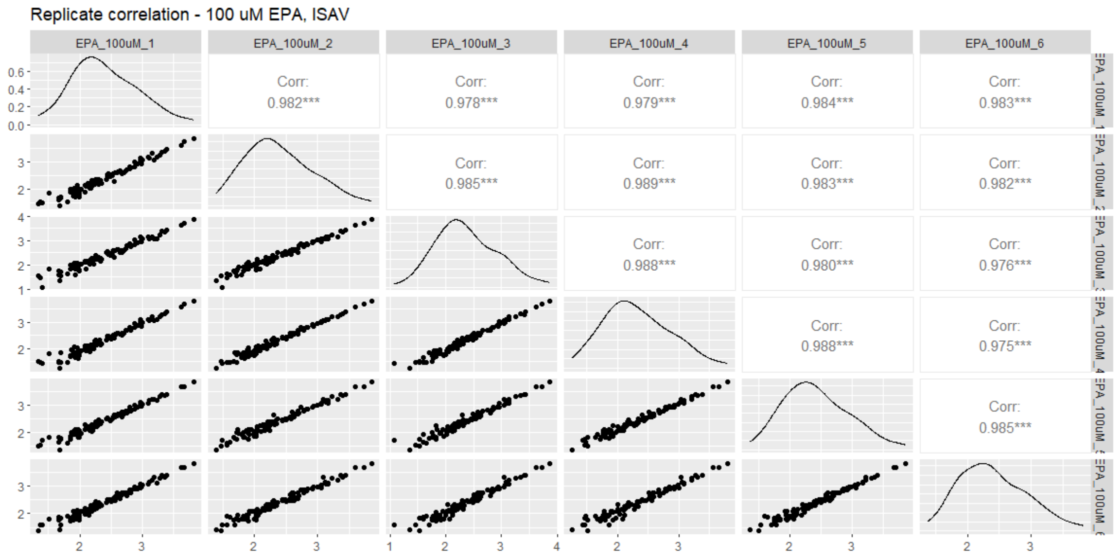

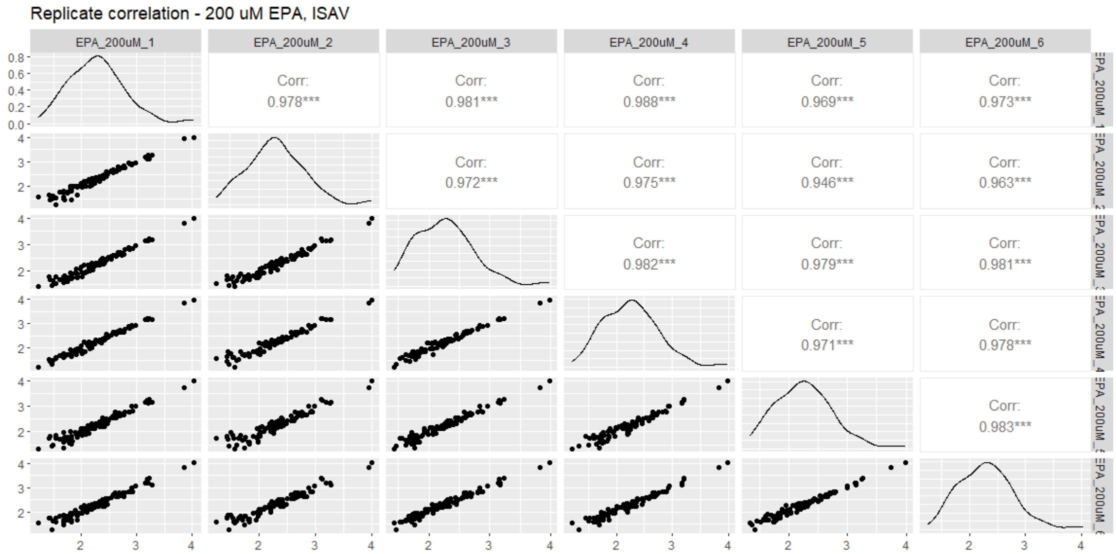

Plots show sample data correlation between 100 randomly selected genes with at least 10 counts in all samples for each experimental group. The panels along the diagonal display the density distribution of counts in the selected samples, and the right side of the panel shows the Pearson correlation coefficient for each sample pair.

Figure S5

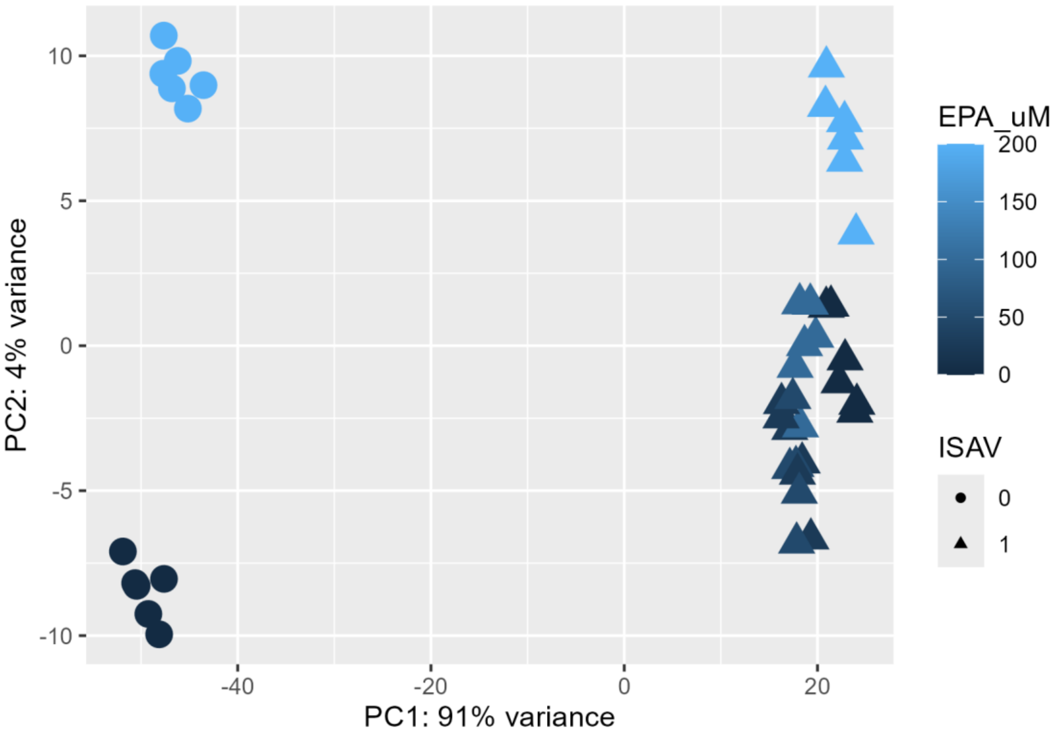

Principal component analysis of VST transformed gene expression values (50 most variable genes) shows that most of the variation in gene expression could be assigned to ISAV. The level of EPA also contributes to variation in the dataset.

Figure S6

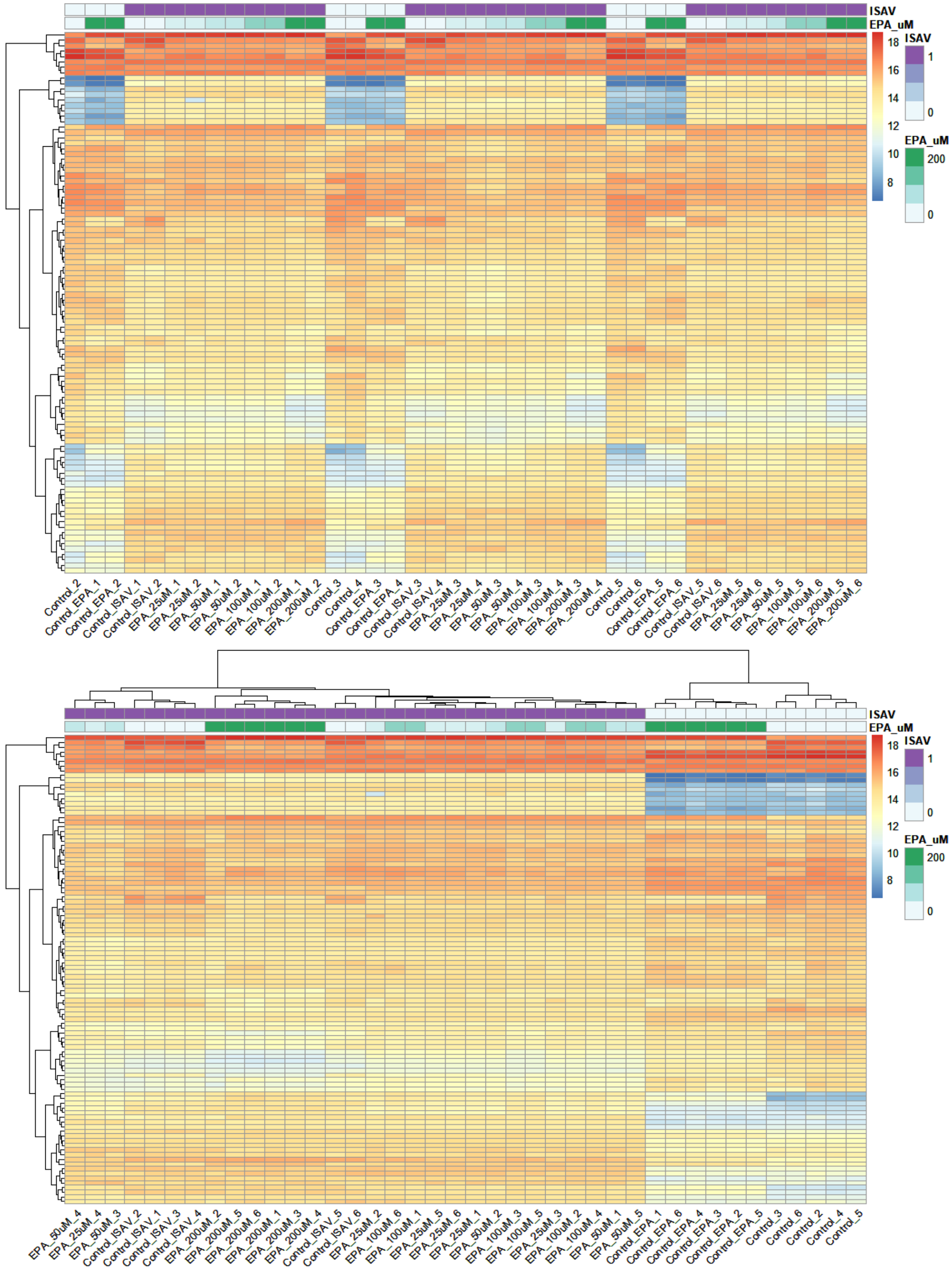

Hierarchical clustering heatmap (euclidian distance) of VST transformed counts from the 100 most variable genes. Upper panel shows the clustering of genes into mainly two groups (sensitive to ISAV). Lower panel also clusters samples into mainly two groups affected by ISAV.

Figure S7

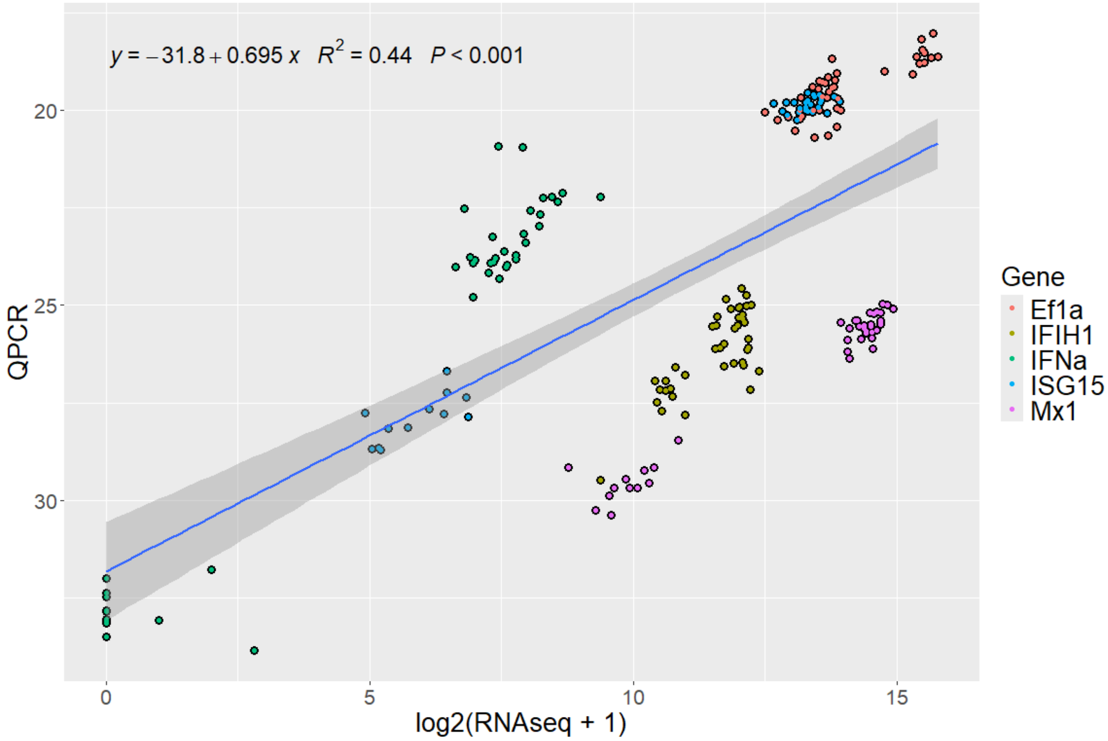

Correlation between qPCR (Ct values) and RNAseq (log2(count+1)) analyses of Atlantic salmon head kidney cells incubated with or without ISAV.

Figure S8

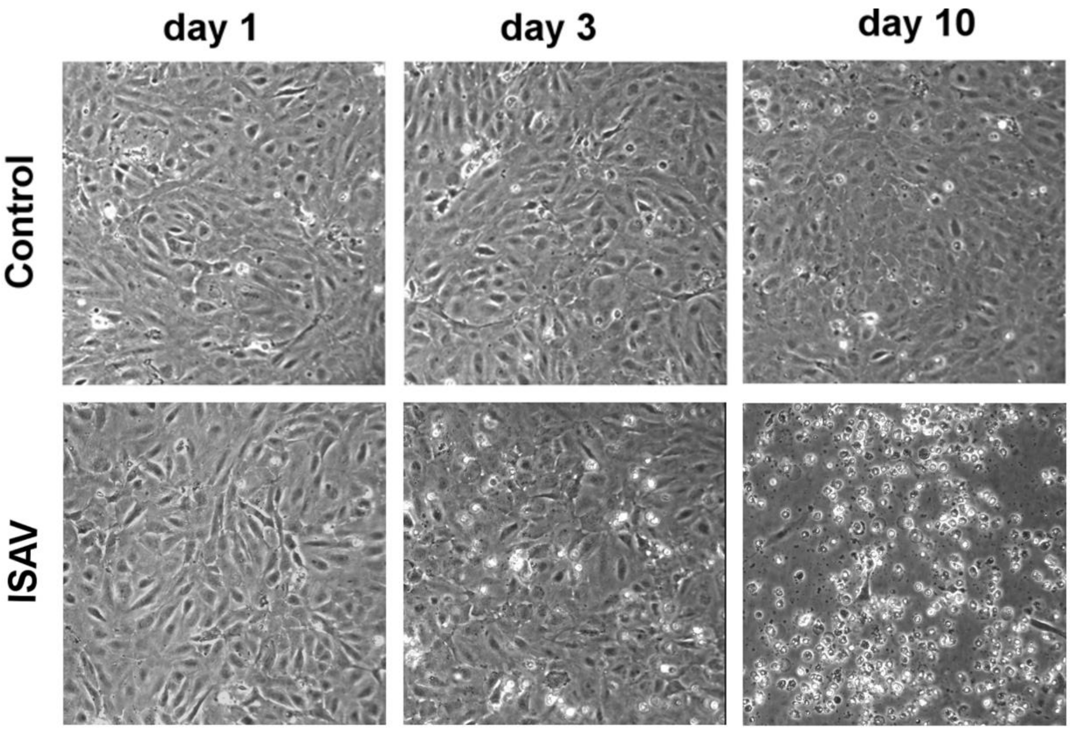

Phase contrast image of Atlantic salmon kidney cells (ASK) at various time points after infection with 1 MOI ISAV. Infected cell culture displays partial (3 days p.i.) and full (10 days p.i.) cytopathic effects.

Session information with list of R packages

```
## R version 4.3.2 (2023-10-31 ucrt)
## Platform: x86_64-w64-mingw32/x64 (64-bit)
## Running under: Windows 10 x64 (build 19045)
##
## Matrix products: default
##
##
## locale:
## [1] LC_COLLATE=Norwegian Bokmål_Norway.utf8
## [2] LC_CTYPE=Norwegian Bokmål_Norway.utf8
## [3] LC_MONETARY=Norwegian Bokmål_Norway.utf8
## [4] LC_NUMERIC=C
## [5] LC_TIME=Norwegian Bokmål_Norway.utf8
##
## time zone: Europe/Oslo
## tzcode source: internal
##
## attached base packages:
## [1] stats4    stats     graphics  grDevices utils     datasets  methods
## [8] base
##
## other attached packages:
##  [1] ggpmisc_0.5.6               ggpp_0.5.7
##  [3] ggsignif_0.6.4              data.table_1.14.10
##  [5] polynom_1.4-1               pheatmap_1.0.12
##  [7] readr_2.1.5                 ggpubr_0.6.0
##  [9] readxl_1.4.3                png_0.1-8
## [11] pander_0.6.5                drc_3.0-1
## [13] MASS_7.3-60.0.1             DESeq2_1.40.2
## [15] SummarizedExperiment_1.30.2 Biobase_2.60.0
## [17] MatrixGenerics_1.12.3       matrixStats_1.2.0
## [19] GenomicRanges_1.52.0        GenomeInfoDb_1.36.4
## [21] IRanges_2.34.1              S4Vectors_0.38.1
## [23] BiocGenerics_0.46.0         genefilter_1.82.1
## [25] factoextra_1.0.7            GGally_2.2.1
## [27] dplyr_1.1.4                 kableExtra_1.4.0
## [29] tidyr_1.3.1                 reshape2_1.4.4
## [31] ggplot2_3.5.1
##
## loaded via a namespace (and not attached):
##  [1] RColorBrewer_1.1-3      rstudioapi_0.15.0       magrittr_2.0.3
##  [4] TH.data_1.1-2           farver_2.1.1            rmarkdown_2.25
##  [7] zlibbioc_1.46.0         vctrs_0.6.5             memoise_2.0.1
## [10] RCurl_1.98-1.14         rstatix_0.7.2           htmltools_0.5.7
## [13] S4Arrays_1.0.5          plotrix_3.8-4           broom_1.0.5
## [16] cellranger_1.1.0        plyr_1.8.9              sandwich_3.1-0
## [19] zoo_1.8-12              cachem_1.0.8            lifecycle_1.0.4
## [22] pkgconfig_2.0.3         Matrix_1.6-5            R6_2.5.1
```

```
## [25] fastmap_1.1.1          GenomeInfoDbData_1.2.10 digest_0.6.34
## [28] colorspace_2.1-0       AnnotationDbi_1.62.2    confintr_1.0.2
## [31] RSQLite_2.3.4          labeling_0.4.3          fansi_1.0.6
## [34] httr_1.4.7             abind_1.4-5             mgcv_1.9-0
## [37] compiler_4.3.2         bit64_4.0.5             withr_2.5.2
## [40] backports_1.4.1        BiocParallel_1.34.2     carData_3.0-5
## [43] DBI_1.2.1              ggstats_0.5.1           highr_0.10
## [46] quantreg_5.97          DelayedArray_0.26.7     gtools_3.9.5
## [49] tools_4.3.2            glue_1.7.0              nlme_3.1-163
## [52] grid_4.3.2             generics_0.1.3          gtable_0.3.4
## [55] tzdb_0.4.0             hms_1.1.3               xml2_1.3.6
## [58] car_3.1-2              utf8_1.2.4              XVector_0.40.0
## [61] ggrepel_0.9.5          pillar_1.9.0            stringr_1.5.1
## [64] vroom_1.6.5            splines_4.3.2           lattice_0.22-5
## [67] survival_3.5-7         bit_4.0.5               annotate_1.78.0
## [70] SparseM_1.81           tidyselect_1.2.0        locfit_1.5-9.8
## [73] Biostrings_2.68.1      knitr_1.45              svglite_2.1.3
## [76] xfun_0.41              stringi_1.8.3           yaml_2.3.8
## [79] evaluate_0.23          codetools_0.2-19        tibble_3.2.1
## [82] cli_3.6.2              xtable_1.8-4            systemfonts_1.0.5
## [85] munsell_0.5.0          Rcpp_1.0.12             XML_3.99-0.16
## [88] parallel_4.3.2         MatrixModels_0.5-3      blob_1.2.4
## [91] bitops_1.0-7           viridisLite_0.4.2       mvtnorm_1.2-4
## [94] scales_1.3.0           purrr_1.0.2             crayon_1.5.2
## [97] rlang_1.1.3            KEGGREST_1.40.1         multcomp_1.4-25
```
